# Single-cell EpiChem jointly measures drug–chromatin binding and multimodal epigenome

**DOI:** 10.1038/s41592-024-02360-0

**Published:** 2024-07-18

**Authors:** Chao Dong, Xiaoxuan Meng, Tong Zhang, Zhifang Guo, Yaxi Liu, Peihuang Wu, Shiwei Chen, Fanqi Zhou, Yanni Ma, Haiqing Xiong, Shaokun Shu, Aibin He

**Affiliations:** 1 Institute of Molecular Medicine, National Biomedical Imaging Center, College of Future Technology, Peking-Tsinghua Center for Life Sciences, Peking University, Beijing, China; 2 State Key Laboratory of Molecular Oncology, Beijing Key Laboratory of Carcinogenesis and Translational Research, Department of Lymphoma, Peking University Cancer Hospital & Institute, Beijing, China; 3 Peking University International Cancer Institute, Beijing, China; 4 Peking University-Yunnan Baiyao International Medical Research Center, Beijing, China; 5 State Key Laboratory of Medical Molecular Biology, Haihe laboratory of Cell Ecosystem, Key Laboratory of RNA and Hematopoietic Regulation, Institute of Basic Medical Sciences, Chinese Academy of Medical Sciences, School of Basic Medicine Peking Union Medical College, Beijing, China; 6 State Key Laboratory of Experimental Hematology, National Clinical Research Center for Blood Diseases, Haihe Laboratory of Cell Ecosystem, Institute of Hematology & Blood Diseases Hospital, Chinese Academy of Medical Sciences & Peking Union Medical College, Tianjin, China; 7 Tianjin Institutes of Health Science, Tianjin, China; 8 Key laboratory of Carcinogenesis and Translational Research of Ministry of Education of China, Peking University Cancer Hospital & Institute, Beijing, China; 9 Peking University Chengdu Academy for Advanced Interdisciplinary Biotechnologies, Chengdu, China

**Keywords:** Biological techniques, Biotechnology

## Abstract

Studies of molecular and cellular functions of small-molecule inhibitors in cancer treatment, eliciting effects by targeting genome and epigenome associated proteins, requires measurement of drug-target engagement in single-cell resolution. Here we present EpiChem for in situ single-cell joint mapping of small molecules and multimodal epigenomic landscape. We demonstrate single-cell co-assays of three small molecules together with histone modifications, chromatin accessibility or target proteins in human colorectal cancer (CRC) organoids. Integrated multimodal analysis reveals diverse drug interactions in the context of chromatin states within heterogeneous CRC organoids. We further reveal drug genomic binding dynamics and adaptive epigenome across cell types after small-molecule drug treatment in CRC organoids. This method provides a unique tool to exploit the mechanisms of cell type-specific drug actions.

## Abstract (web summary)

EpiChem enables the co-profiling of small molecule–DNA interactions and multiple epigenomic features in single cells.

## Main

Small molecules play a role in regulating cellular processes, and impacting gene expression, chromatin structure and signaling pathways. These interactions serve as the foundation for a wide array of therapeutic interventions, encompassing both conventional pharmaceuticals and advanced targeted therapies^1–4^. Although the efficacy of numerous small chemical molecule drugs is well established in medical treatments through interactions with the genome, the specific mechanisms underlying genomic interactions often remain unclear^5–7^. Mapping small molecules that bind DNA or chromatin associated proteins is fundamental to decipher how they function in anticancer treatment.

Until now, only a few techniques have been reported to measure the interaction between small molecules and cellular DNA in bulk samples, and these often require millions of cells. Chem-seq and Click-Chem-seq, for instance, detect genomic binding of small molecules by utilizing an affinity tag to enrich sheared chromatin bound by therapeutic drugs^8,9^. Chem-map^10^ enables mapping of these interactions by target tagmentation in a biochemical design as widely used for profiling chromatin proteins–DNA interaction^11–15^; however, single-cell profiling of small molecules-target engagement has never been attained thus far. In addition, single-cell co-assay of drug genomic binding and the epigenetic landscape is also of pivotal importance to illustrate the compatibility of drug targets and epigenetic context. By allowing an in-depth exploration of cellular heterogeneity within populations within the tumor microenvironment, single-cell techniques may unveil nuanced variations in drug responses. The knowledge of links between drug engagement and epigenetic landscape at single-cell level would provide a comprehensive view of the drug response and functional heterogeneity and molecular mechanisms^6,16–26^.

Here we introduce scEpiChem, a method enabling single-cell simultaneous detection of small molecule drug-target engagement and epigenetic profiles. The methodology involves the incubation of biotinylated (btn) small molecules with anti-biotin antibody, combined with the barcoded protein A-Tn5 (PAT) transposase-mediated tagmentation process to selectively profile specific genomic regions within individual cells. Utilizing several rounds of combinatorial barcoding, scEpiChem allows for retrieval of hundreds of thousands single-cell profiles in a single experiment, which is scalable up to millions of single cells. With an emphasis on capturing the nuances of cellular heterogeneity, scEpiChem represents an approach to dissecting the intricate interplay between small molecules and genomic elements in single cells.

## Results

### scEpiChem enables high-throughput profiling of genomic binding sites of small-molecule drugs

**Fig. 1:**
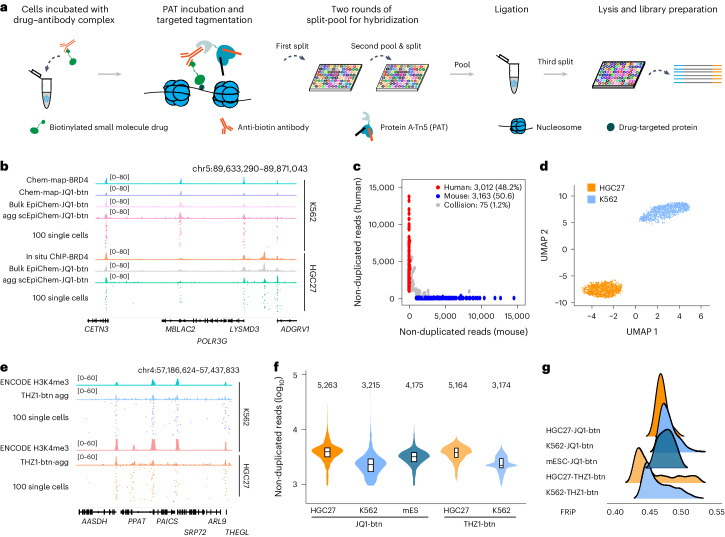
Single-cell EpiChem profiling of genomic binding sites of small-molecule drugs. **a**, Schematic of single-cell EpiChem design. **b**, Track view displaying JQ1-btn signals in K562 and HGC27 cells at the representative loci. Genomic tracks of JQ1-btn binding in bulk level (JQ1-btn), single cell aggregate (JQ1-btn agg) and randomly selected 100 single cells alongside those of related proteins were presented. Chem-map data were downloaded from GSE209713. **c**, Scatter-plot of the human–mouse species mixing test using JQ1-btn signals. Points are colored by the cell identity as human (red, >95% of reads mapping to hg19) and mouse (blue, >95% of reads mapping to mm10) or collision (gray, 5% to 95% of reads mapping to either genome). **d**, UMAP visualization showing K562 (*n* = 2,000) and HGC27 cells (*n* = 2,000) colored by cell types, based on JQ1-btn signals. **e**, Track view displaying THZ1-btn signals in K562 and HGC27 cells at the representative loci. Genomic tracks of small-molecule genomic bindings at bulk level (THZ1-btn), single cell aggregate (THZ1-btn agg) and randomly selected 100 single cells alongside those of related proteins were presented. **f**, Violin plot showing non-duplicated reads per cell of JQ1-btn and THZ1-btn of K562 (*n* = 2,000), HGC27 (*n* = 2,000) and mES cells (*n* = 2,000). Numbers on the top of violin plot indicate the median value. **g**, Ridge plot showing the FRiP of JQ1-btn, THZ1-btn in K562, HGC27 and mES cells. Source data

**Extended Data Fig. 1:**
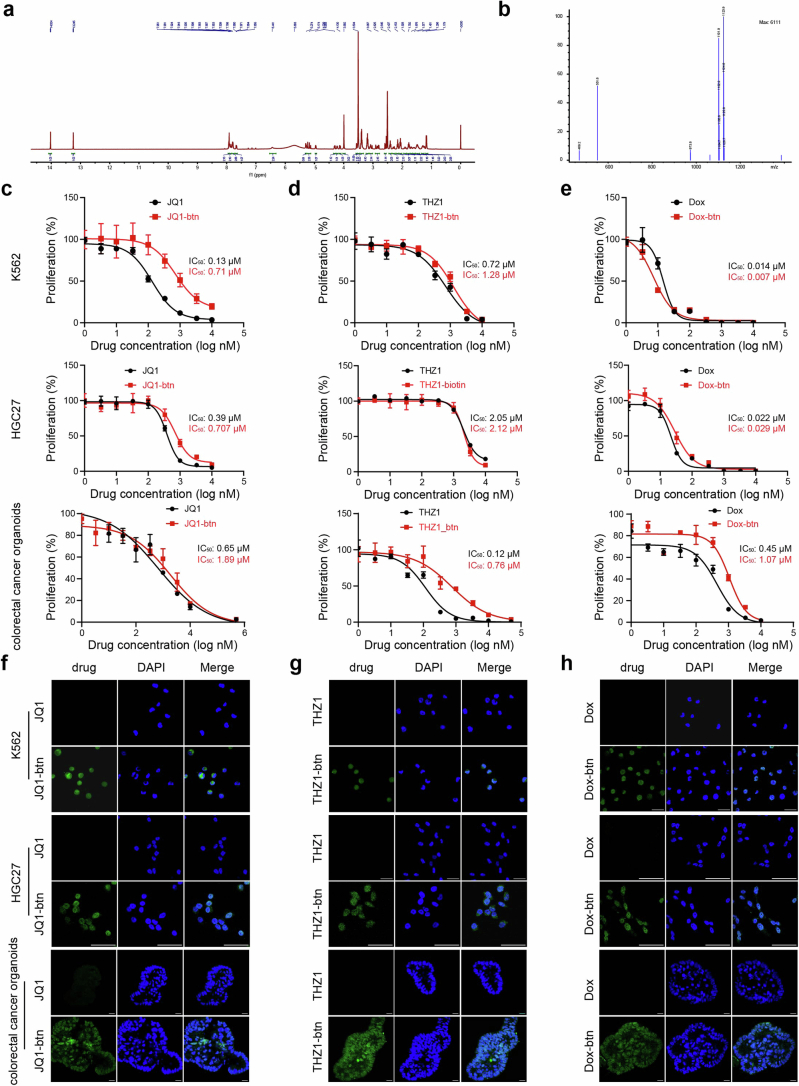
Characterization of native and biotinylated small molecules. **a** ^1^H NMR spectrum (400 MHz) of Doxorubicin-btn (Dox-btn) in DMSO solvent. **b** Mass spectrum of Dox-btn. **c-e** Effect of different native small molecules and biotinylated derivatives on K562 (top), HGC27 (middle) and colorectal cancer organoids (bottom) proliferation. Cells were treated with varying concentrations of JQ1 and JQ1-btn (**c**), THZ1 and THZ1-btn (**d**), and Dox and Dox-btn (**e**). Mean ± s.d. (n = 3). **f-h** Confocal microscopy of K562 (top), HGC27 (middle) and colorectal cancer organoids (bottom) treated with different small molecules and biotinylated derivatives. Cells were stained for JQ1 and JQ1-btn (**f**), THZ1 and THZ1-btn (**g**), and Dox and Dox-btn (**h**) by the anti-biotin antibody. Nuclei were stained with DAPI. Scale bars, 20 μm (**f**, **g**, **h**). Shown are one of experiments repeated at least three times.

**Extended Data Fig. 2:**
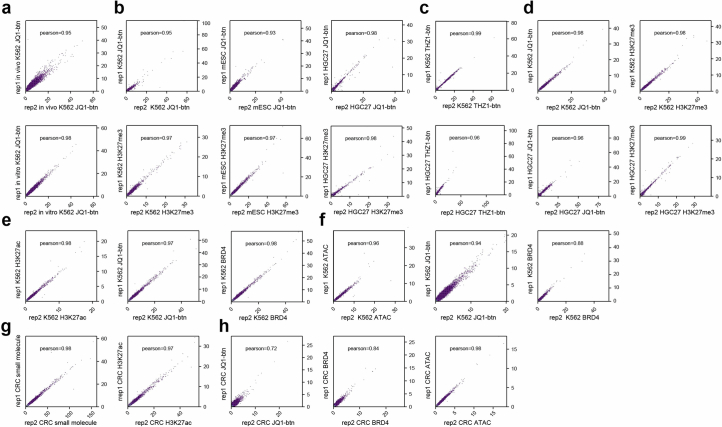
Data reproducibility of EpiChem and scEpiChem experiments. **a-h** Scatter plot showing correlation between biological replicates in EpiChem (**a**), scEpiChem (single modality: JQ1-btn) in K562, mESC and HGC27 (**b**), scEpiChem (single modality: THZ1-btn) in K562 and HGC27 (**c**), scEpiChem (dual modalities: JQ1-btn and H3K27me3) in K562 and HGC27 (**d**), scEpiChem (tri-modality: JQ1-btn, BRD4 and H3K27ac) in K562 (**e**), scEpiChem (tri-modality: JQ1-btn, BRD4 and ATAC) in K562 (**f**), scEpiChem (dual modalities: H3K27ac and small molecule) in CRC organoids (**g**) and scEpiChem (tri-modality: JQ1-btn, BRD4 and ATAC) in CRC organoids (**h**). Each replicate represents one individual experiment.

**Extended Data Fig. 3:**
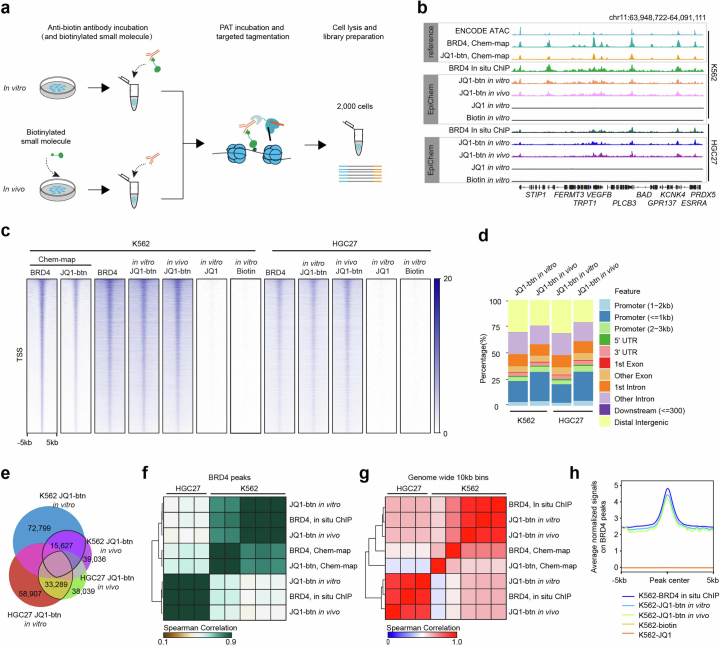
Characterization of EpiChem profiling of genomic binding sites of the BET bromodomain-targeting drug JQ1-btn in K562 and HGC27 cells. **a** EpiChem workflow. **b** Track view displaying EpiChem signals of JQ1-btn in K562 and HGC27 cells, on the representative loci of cell-type specific drug binding sites (*FERMT3*). BRD4 (*in situ* ChIP) and JQ1-btn (*in vitro* and *in vivo* experiments) data were obtained in this work, alongside JQ1 and biotin as two negative controls. Chem-map data were downloaded from GSE209713. **c** Heatmap showing EpiChem signals around Chem-map BRD4 peak regions (51,963). The rows were sorted by the descending signals of Chem-map BRD4 signals. **d** Genomic distributions of JQ1-btn peaks from EpiChem *in vitro* and *in vivo* experiments. Total peaks number: K562-JQ1-btn *in vitro*: 72,799; K562-JQ1-btn *in vivo*: 39,036; HGC27-JQ1-btn *in vitro*: 58,907; HGC27-JQ1-btn *in vivo*: 38,039. **e** Venn diagram showing peak overlap in (**d**) of JQ1-btn (*in vitro* and *in vivo*) in K562 and HGC27 cells. Overlap peak numbers: K562 JQ1-btn *in vivo* and *in vitro*: 15,627; HGC27 JQ1-btn *in vivo* and *in vitro*:33,289; K562 JQ1-btn *in vitro* and HGC27 JQ1-btn *in vitro*: 19,288; K562 JQ1-btn *in vitro* and HGC27 JQ1-btn *in vitro*: 22,872. **f** Spearman correlation of JQ1-btn (*in vitro* and *in vivo*) and BRD4 (*in situ* ChIP) binding signals within the Chem-map BRD4 peak regions (51,963) in K562 and HGC27 cells. **g** Spearman correlation of JQ1-btn (*in vitro* or *in vivo*) or BRD4 binding signals in K562 and HGC27 cells, in 10 kb genome wide. **h** Average JQ1-btn (*in vitro* and *in vivo*), JQ1 and biotin signals in K562 cells were plotted at the 5 kb flanking regions around the peak center of BRD4 from Chem-map (51,963).

scEpiChem (Fig. 1a) utilizes split-pool barcoding^27–36^ and protein A-Tn5-antibody-directed target tagmentation to acquire single-cell small-molecule binding sites. In brief, (1) slightly fixed and permeabilized cells were incubated with a complex of biotinylated small molecules and anti-biotin antibody; (2) cells were tagmented by six pre-assembled protein A-Tn5 with T5 barcoded adaptors (PAT-T5) (Supplementary Table 1) to label specific genomic regions targeted by small molecules; (3) cells with transposed chromatin fragments were distributed into 96-well plates for two rounds of hybridization with specific barcoded oligonucleotides (Supplementary Table 1); (4) cells were pooled for ligation and subsequently redistributed to 1,000–3,000 cells per well for the fourth round of indexed PCR using 96 barcoded oligonucleotides; (5) subsequently, DNA fragments were amplified and sequenced (see also Methods and Supplementary Protocol). We first validated the bioactivity of these small-molecule drugs, the BET bromodomain protein inhibitor JQ1 and its biotinylated derivative JQ1-btn, CDK7 inhibitor THZ1 and THZ1-btn, and the topoisomerase-II inhibitor doxorubicin (Dox) and Dox-btn in both human leukemia K562 and gastric cancer HGC27 cells as well as human CRC organoids (Extended Data Fig. 1a–h). Compared to Chem-map data using hundreds of thousands of cells, we achieved comparable data quality with JQ1-btn binding sites by EpiChem with 2,000 cells in both in vivo and in vitro experiments (Extended Data Fig. 2 and Supplementary Table 2). It has been reported that JQ1 binds to BET proteins, including BRD2, BRD3 and BRD4. BET proteins share the majority of genomic binding sites, whereas JQ1 occupancy of chromatin is most strongly correlated with that of BRD4 than other BET proteins^37^. We also confirmed the expected highest overlap between JQ1-btn and its protein target BRD4 as well as cell type-specific binding sites (Extended Data Fig. 3).

**Extended Data Fig. 4:**
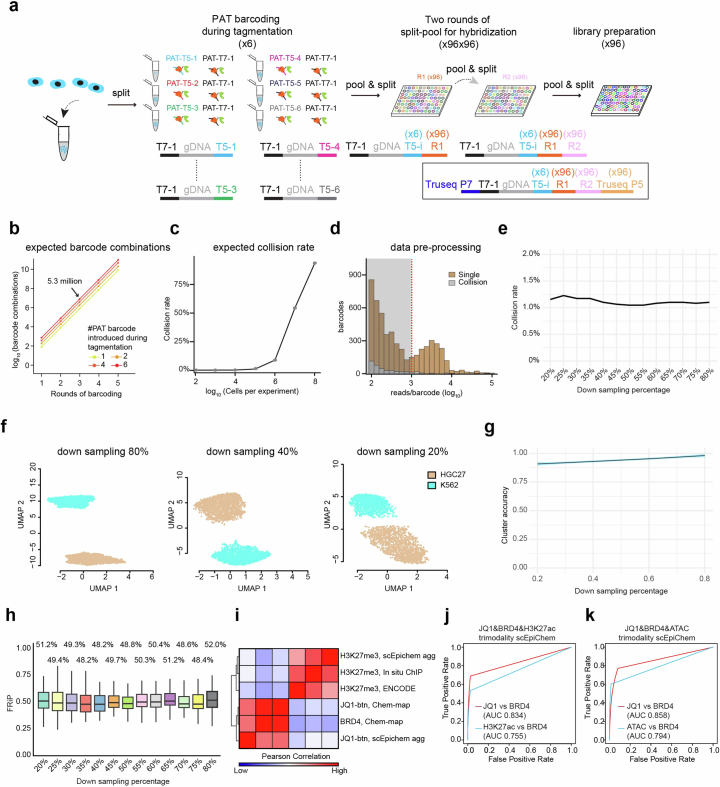
Single-cell EpiChem simultaneously detects JQ1–BRD4 engagement and chromatin accessibility/H3K27me3. **a** The structure of scEpiChem sequencing library. **b** The expected number of barcode combinations using different numbers of PAT barcode introduced during tagmentation. The arrow indicated the expected barcode combinations in our scEpiChem experiments. **c** Expected collision rate for different cells per experiment in scEpiChem. **d** Read distribution for human/mouse mix-species scEpiChem data. The red dashed line indicates 1,000 non-duplicated reads, and barcodes with less than 1,000 reads were excluded from further analyses. **e** The broken line plot shows collision rate in the human-mouse species mixing test by scEpiChem single modality (JQ1-btn) data. **f** Benchmarking the ability of clustering of scEpiChem data with different sequencing depth. Box plots show the number of ‘Mixed’ cells occupying non-ambiguous clusters defined exclusively by either K562 or HGC27 cells in 80%, 40%, 20% down sampled reads. **g** The cluster accuracy of scEpiChem varied with down sampling reads. The line graph illustrates the change in the cluster accuracy, defined exclusively by either K562 or HGC27 cells in 80%, 40%, 20% down sampling reads. Shaded error band shows the 95% confidence interval (mean ± 2 SEM). **h** The boxplots show the number of single-cell detected reads in bulk peaks with scEpiChem after down sampling the reads of the single modality data from 20% (left) to 80% (right) (n = 2,000). **i** Heatmap showing Pearson correlation of aggregated single-cell genome-wide signals of JQ1-btn and H3K27me3 of dual modalities scEpiChem data in 2,000 K562 cells, and JQ1-btn or BRD4 signals from Chem-map, or H3K27me3 signals from ENCODE data. **j-k** ROC curves for aggregate JQ1&BRD4&H3K27ac (**j**) and JQ1&BRD4&ATAC (**k**) tri-modality scEpiChem data in K562 cells. Values next to indicated group names are the area under the ROC (AUC). The aggregate BRD4 data serves as the gold standard for specificity analysis.

As a proof of concept, we validated the efficacy of scEpiChem using JQ1-btn in a 1:1:2 mixture of human K562 and HGC27 cells and mouse embryonic stem (mES) cells. Both aggregate and single cells profiles of JQ1-btn in K562 and HGC27 cells exhibited similar patterns with BRD4 around representative genomic loci, but showing differential JQ1-btn binding sites between the two human cell lines (Fig. 1b). Human and mouse reads were well separated on chromatin profiles, representing an expected collision rate (1.2%) (Fig. 1c), close to the theoretical collision rate of 0.9% (Extended Data Fig. 4a–d). Next, we investigated whether the observed collision rate could be influenced by different sequencing depths. After downsampling of sequencing reads, single-cell data with JQ1-btn showed no detected difference (Extended Data Fig. 4e). To demonstrate the performance of scEpiChem in distinguishing cell types, we applied Uniform Manifold Approximation and Projection (UMAP) to reduce the dimensionality of K562 and HGC27 (Fig. 1d). Similarly, we confirmed that there was no discernible difference in both cluster accuracy and single-cell detected reads in bulk peaks as the depth of sequencing varied (Extended Data Fig. 4f–h). Finally, we also profiled the THZ1-btn in K562 and HGC27 (Fig. 1e). Consistent data quality in non-duplicated reads and the fraction of reads in peaks (FRiP) for datasets from two different small molecules, JQ1-btn and THZ1-btn, was obtained (Fig. 1f,g). To assess library complexity, we performed deep sequencing, yielding PCR duplication rates of 83.12% for JQ1-btn in K562 and HGC27 cells and 85.56% for JQ1-btn in mES cells (Supplementary Table 3). After quality control filtering, we determined that the median non-duplicated reads per cell for JQ1-btn was 5,263 in K562, 3,215 in HGC27 and 4,175 in mES cells. The median non-duplicated reads per cell for THZ1-btn was 5,164 in K562 and 3,174 in HGC27. Taken together, our results indicated that scEpiChem enables high-throughput profiling of genomic binding sites targeted by small molecules with a high signal-to-noise ratio. Despite high signal-to-noise data by scEpiChem as demonstrated by FRiP, further optimization experiments may help increase the reads per cell by reducing the duplication rate.

### scEpiChem enables joint assay of small-molecule JQ1-btn genomic binding and histone modification or chromatin accessibility

**Fig. 2:**
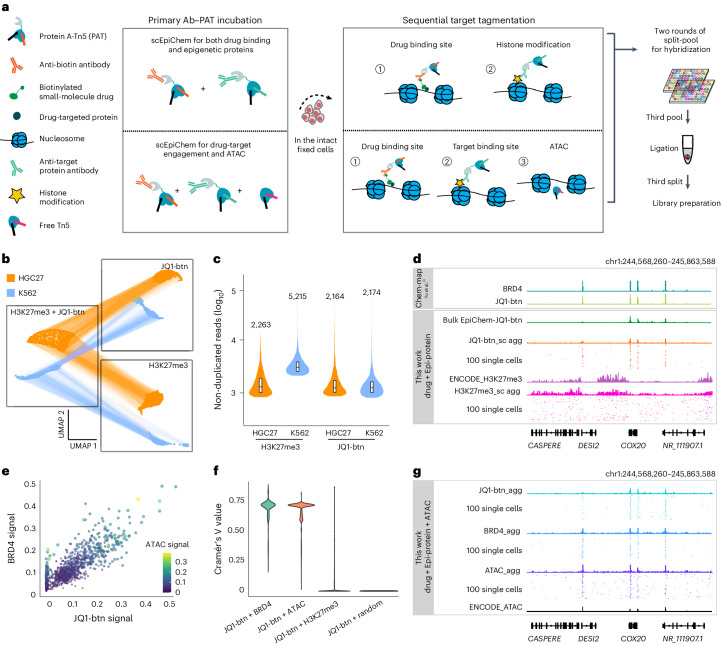
Single-cell EpiChem enables joint assay of small-molecule JQ1 genomic binding and histone modification or chromatin accessibility. **a**, Schematic of single-cell EpiChem design for the joint assay. **b**, UMAP embedding of dual modalities of scEpiChem data for JQ1-btn and H3K27me3. Connecting lines represent the same cells in different modalities (3,619 cells in two biological replicates). **c**, Violin plot showing the median non-duplicated reads of JQ1-btn and H3K27me3 of K562 (*n* = 2,000) and HGC27 cells (*n* = 2,000). Boxes in the violin plots: center marks the median and edges of boxes define the 25th and 75th percentiles. **d**, Track view of aggregate single cells of scEpiChem (dual modalities) K562 data and random 100 single cells with bulk reference, at the *DESI2* loci. Chem-map data were downloaded from GSE209713; ENCODE H3K27me3 were downloaded from GSE31755. **e**, Visualization of single-cell ATAC, BRD4, JQ1-btn modality of scEpiChem tri-modality data in K562 cells (*n* = 1,261). Normalized signal was calculated by JQ1-btn, BRD4 and ATAC read counts in 56,352 BRD4 peaks and then *z*-score normalized. **f**, Violin plots depicting the calculated Cramér’s V of association between JQ1-btn & BRD4 modalities (*n* = 1,261, median 0.71), JQ1-btn & ATAC (*n* = 1,261, median 0.65), JQ1-btn & H3K27me3 (*n* = 3,619, median 0.07) and JQ1-btn & random (*n* = 1,261, median 0.03). **g**, Track view of aggregated single cells of scEpiChem (tri-modalities) data and random 100 single cells with bulk reference from ENCODE at the *DESI2* loci. ENCODE ATAC were downloaded from GSE90409. Source data

We next expanded the capability of scEpiChem by incorporating sequential tagmentation to enable joint profiling of both small-molecule genomic binding and histone modifications within individual cells (Fig. 2a). This approach may reveal potential therapeutic targets and their interplay with multimodal epigenomic landscape. Specific primary antibodies against anti-biotin antibody (together with biotinylated small molecules) or histone modification were incubated with pre-assembled protein A-Tn5 with different T7 barcoded adaptor (PAT-T7). The complexes of primary antibody–PAT-T7 were directed to targeted chromatin and sequential tagmentation occurred. The distinct identities of the antibody were identified through corresponding T7 barcodes and deconvoluted during the data processing. Similar to the validation in single modality profiling, the ability of scEpiChem to distinguish a mixture of cells in dual modalities was confirmed using JQ1-btn in a 1:1 mixture of human K562 and HGC27 cells. We projected single-cell data into low-dimensionality space based on features defined by H3K27me3, JQ1-btn or a combination of both using weighted-nearest neighbor integration^38^, and clustered the resulting single cells, yielding 0.02% (10 out of 3,619) ‘mixed’ cells occupying non-ambiguous clusters defined exclusively by either K562 or HGC27 cells (Fig. 2b). The median non-duplicated reads per cell was 5,215 for H3K27me3 and 2,263 for JQ1-btn in K562 cells, whereas this was 2,174 for H3K27me3 and 2,164 for JQ1-btn in HGC27 cells (Fig. 2c). To further globally evaluate data quality of scEpiChem, we calculated the correlation based on the signals at 5-kb bins genome wide. We found that aggregate single-cell JQ1-btn profiles were highly correlated with bulk JQ1-btn and BRD4 from the chem-map dataset, and there was a clear separation between JQ1-btn and H3K27me3 (Extended Data Fig. 4i). Similarly, we observed aggregate single-cell JQ1-btn signals around representative BRD4 loci, whereas H3K27me3 and JQ1-btn signals are mutually exclusive (Fig. 2d). This discovery aligns with the knowledge that BRD4, the target of JQ1-btn, engages with chromatin regions characterized by hyperacetylated histones, lacking H3K27me3.

**Extended Data Fig. 5:**
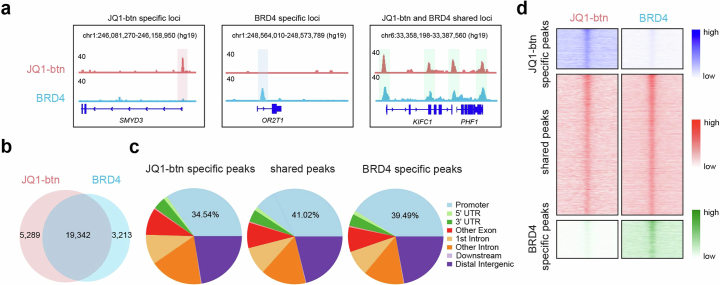
The discrepancies of binding site of JQ1-btn and its target BRD4 were revealed through scEpiChem data in K562 cells. **a** Track view of JQ1-btn and BRD4 signals on the representative loci in K562 cells. JQ1-btn specific loci (left), BRD4 specific loci (middle) and JQ1-btn and BRD4 shared loci (right). **b** Venn diagram showing peak overlap of JQ1-btn and BRD4 in K562 cells. Overlap peak numbers of JQ1-btn and BRD4: 19,342; JQ1-btn specific peak numbers: 5,289; BRD4 specific peak numbers: 3,213. **c** Genomic distributions of scEpiChem peaks of JQ1-btn and BRD4 in K562 cells. **d** Heatmap showing JQ1 and BRD4 binding signals of scEpiChem data in JQ1-specific (top), shared (middle) and BRD4-specific (bottom) peaks in K562 cells.

In pursuit of simultaneously identifying cellular identities and assessing drug-target engagement at the single-cell level, we introduced chromatin accessibility profiling module by scATAC-seq into scEpiChem. In our initial experiments with the K562 cell line, we concurrently captured signals from JQ1-btn, BRD4 and ATAC. The complex of JQ1-btn with the anti-biotin antibody or BRD4 antibody was pre-incubated separately with different PAT-T7 complexes. After sequential incubation of antibody–PAT for targeted tagmentation, different PAT-T5 complexes conjugated with secondary antibody were then introduced. Finally, ATAC labeling was performed using Tn5-T7 and Tn5-T5. Demultiplexing different T7 barcodes yielded reads for three distinct omics datasets. To illustrate the compatibility of the three modalities, we calculated the normalized signals of JQ1-btn, BRD4 and ATAC on the BRD4 peaks (Fig. 2e). Expectedly, the genomic binding signals of JQ1-btn have a strong correlation with BRD4. Next, we calculated Cramér’s V^28^ to quantify the degree of co-enrichment between each pair of targets in the same single cells, revealing a slightly higher degree of co-enrichment between JQ1-btn and BRD4 (71%) than JQ1-btn with ATAC (65%), markedly higher than with H3K27me3 (7%) or random (2%) (Fig. 2f). Likewise, we detected aggregate single-cell JQ1-btn signals around representative BRD4 loci (Fig. 2g). For the specificity assessment, we conducted receiver operating characteristic (ROC) analysis, revealing an overall similarity but also discrepancy between drug binding and active/accessible chromatin regions (Extended Data Fig. 4j,k). To further discern the subtle differences between JQ1-btn and its target BRD4, we performed additional analyses with JQ1-btn–BRD4 scEpiChem data in K562 cells (Extended Data Fig. 5). Beyond the overall similarity between JQ1-btn and BRD4, BRD4- and JQ1-specific signals were expectedly identified and visualized at representative gene loci (Extended Data Fig. 5a). We identified 5,289 JQ1-specific, 3,213 BRD4-specific and 19,342 shared peaks between JQ1 and BRD4 scEpiChem data in K562 cells (Extended Data Fig. 5b). Compared to the shared peaks between JQ1-btn and BRD4 profiling, JQ1-btn-specific peaks had a slightly lower proportion of reads mapping to promoter regions (Extended Data Fig. 5c). Furthermore, a heatmap revealed specific JQ1-btn and BRD4 signals in K562 cells (Extended Data Fig. 5d). Therefore, EpiChem is able to achieve identification of subtle differences between a drug and its target protein binding. Taken together, our results indicate that scEpiChem enables successful simultaneous measurement of drug-target engagement and chromatin binding proteins/accessibility.

### Integrated multimodal analysis reveals diverse drug interactions with tumor heterogeneity

**Fig. 3:**
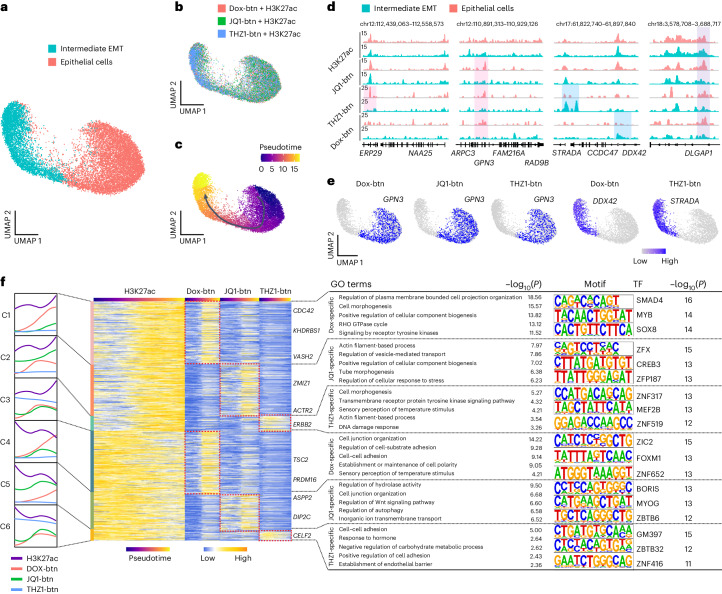
Single-cell EpiChem identifies cell type-specific genomic binding dynamics of three small-molecule drugs in human CRC organoids. **a**, UMAP showing scEpiChem (small molecules and H3K27ac) in human CRC organoids (*n* = 14,027), identified as epithelial cells (*n* = 9,341) and Intermediate EMT cells (*n* = 4,686). **b**, UMAP showing undetected batch effects in different scEpiChem experiments (small molecules and H3K27ac) in CRC organoids (*n* = 14,027), visualizing Dox-btn + H3K27ac (*n* = 4,793), JQ1-btn + H3K27ac (*n* = 4,810) and THZ1-btn + H3K27ac (*n* = 4,424). **c**, Pseudotime trajectory showing EMT progression. **d**, Track view displaying signals of H3K27ac and three small molecules in epithelial and intermediate EMT cells on the representative loci of cell type-specific drug binding sites. The pink, blue and purple shading represents epithelial cell-specific, intermediate EMT-specific and common peaks, respectively. **e**, UMAP projections showing gene activity scores of *GPN3*, *DDX42* and *STRADA* among small molecules. **f**, Aggregate curves (left) and heatmaps (middle) showing dynamic genomic signals of H3K27ac and small molecules (Dox-btn, JQ1-btn and THZ1-btn) along the pseudotime. Representative genes in each cluster were labeled on the right. The top three enriched GO terms in each cluster are shown (right). De novo transcription factor motifs in peaks were discovered using Homer. *P* values were calculated by the Binomial test. The results of GO term enrichment analysis using a hypergeometric test, with two-sided statistical tests and adjustments for multiple comparisons employing the Benjamini–Hochberg method. Source data

**Extended Data Fig. 6:**
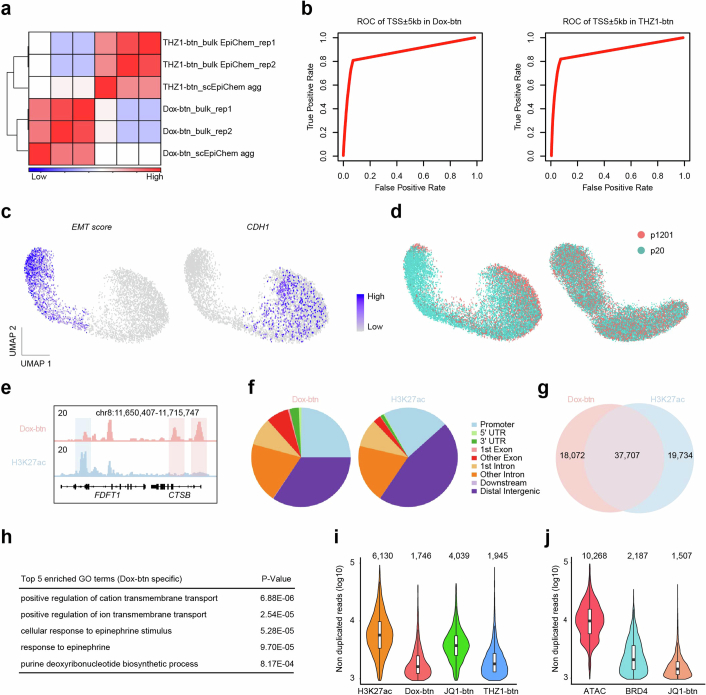
Characterization of scEpiChem data in human CRC organoids. **a** Spearman correlation of THZ1-btn and Dox-btn binding signals in bulk and single cell data within the TSS 5±kb regions in human CRC organoids. **b** ROC curves for CRC bulk and single cell aggregated THZ1-btn (4,424 cells) or Dox-btn (4,793 cells), using CRC bulk THZ1-btn or Dox-btn data as gold standard. AUC: THZ1-btn, 0.9561; Dox-btn, 0.8361. **c** UMAP showing scEpiChem data (small molecules and H3K27ac) on CRC organoids (n = 14,027 cells) with projections of the gene activity scores of the EMT score and CDH1. The EMT score was calculated based on 1,184 signature genes (http://www.dbemt.bioinfo-minzhao.org/). **d** UMAP showing the batch effect of scEpiChem (small molecules and ATAC; small molecules, target and ATAC) on CRC organoids data from two samples, p1201 (n = 6,209) and p20 (n = 3,222); p1201 (n = 12,790) and p20 (n = 8,993). **e** Track view of H3K27ac and Dox-btn signals on the representative loci in CRC organoid cells. **f** Genomic distributions of scEpiChem peaks of Dox-btn and H3K27ac in CRC organoid cells. **g** Venn diagram showing peak overlap of Dox-btn and H3K27ac in CRC organoid cells. **h** GO terms enriched by Dox-btn-specific peaks. P-value was calculated by binomial test. The results of GO term enrichment analysis using a hypergeometric test, with two-sided statistical tests and adjustments for multiple comparisons employing the Benjamini-Hochberg method. **i-j** Distribution of the median non-duplicated reads for single cells in H3K27ac (n = 12,066), Dox-btn (n = 3,052), JQ1-btn (n = 4,012) and THZ1-btn (n = 3,453) with scEpiChem (small molecules and H3K27ac) (**i**) and ATAC (n = 10,672), BRD4 (n = 5,809) and JQ1-btn (n = 5,302) with scEpiChem (small molecules, target and ATAC) (**j**) in CRC organoids data. Boxes in the violin plots: center marks the median; edges of boxes define the 25th and 75th percentiles.

The process of epithelial–mesenchymal transition (EMT) plays a critical role in various biological phenomena, including embryonic development and cancer metastasis^39–41^. To characterize the dynamic genomic bindings of small molecules in this context, we performed scEpiChem with two patient-derived CRC organoids by measuring H3K27ac (marking putative active enhancers) and three small molecules (JQ1-btn, 4,810 cells; THZ1-btn, 4,424 cells; Dox-btn, 4,793 cells). After quality control, 14,027 single cells were retained for further analyses (Extended Data Fig. 6a–d). We first visualized the Dox-btn- and H3K27ac-specific and shared signals at representative loci (Extended Data Fig. 6e). Compared to Dox-btn, H3K27ac profiles were more enriched at distal enhancer regions (Extended Data Fig. 6f). Gene Ontology (GO) term enrichment analysis revealed that Dox-specific features were involved in positive regulation of ion transmembrane transport and cellular metabolic processes (Extended Data Fig. 6h), consistent with the previously reported Dox function. Clustering single cells by H3K27ac profiles identified two major populations, annotated as epithelial (9,341) and intermediate EMT cells (4,686) (Fig. 3a,b and Extended Data Fig. 6i). Next, we examined how small-molecule binding sites changed in association with H3K27ac during EMT progression. Initially, we investigated a potential lineage relationship between the distinct cell states present in the CRC organoids using pseudotime analyses. The result suggested a hypothetical differentiation trajectory from epithelial to intermediate EMT cells (Fig. 3c). Moreover, we detected variations in small-molecule binding as epithelial cells transitioned into intermediate EMT cells. By analyzing the signal from aggregated single cells, we observed the signal enrichment of JQ1-btn, THZ1-btn and Dox-btn on *GPN3* during EMT (Fig. 3d,e). To investigate the possibility of different targets of small molecules in deconvolution of cell types, we performed dimensionality reduction analysis using the JQ1-btn (4,810 cells), THZ1-btn (4,424 cells) and Dox-btn (4,793 cells) profiles separately. We selected 11,154 H3K27ac peak regions overlapping with either of these small-molecule binding sites. Using *k*-means clustering, we categorized these regions into six groups with differential small molecules genomic distributions along the dynamic trajectory of H3K27ac signals from epithelial to intermediate EMT cells (Fig. 3f). Cluster 1–3 (C1–C3) regions were associated with cooperative increases in small-molecule and H3K27ac signals (Dox-btn, 26.0% of all tested regions; JQ1-btn, 19.4% of all tested regions; THZ1-btn, 6.5% of all tested regions). We conducted GO analysis and delineated unique functional categories of genes within each group (Fig. 3f). For example, genes in C1 were strongly enriched for RHO GTPase cycle, including genes related to small GTP binding protein such as Cell Division Cycle 42 (*CDC42*) gene. Cdc42, through its connection to the adherens junction via IQGAP1, seems to exert a contrasting effect on junctional stability^42,43^. Our data suggest that genes related to small GTP binding protein are activated by similar mechanisms in the H3K27ac and Dox-btn enriched regions during EMT. Genes in C2 and C3 were strongly enriched for actin filament-based processes, including actin-related genes (Actin Related Protein 2, *ACTR2*). C4–C6 regions exhibited distinct patterns between the small-molecule and H3K27ac signals (Dox-btn, 27.6% of all tested regions; JQ1-btn, 16.2% of all tested regions; THZ1-btn, 4.3% of all tested regions). GO analysis revealed that C4 and C5 were enriched for terms of cell junction organization, whereas C6 genes were enriched for terms, including establishment of endothelial barrier. We conducted de novo transcription factor motif enrichment analysis on these drug-specific peaks, uncovering enrichment of TFs such as SMAD4, ZFX and ZNF317 for Dox-btn, JQ1-btn and THZ1-btn, respectively, providing insights into potential affected downstream transcription factor targets. Together, our results reveal the differential, dynamic genomic binding sites of these small molecules during human CRC organoid cultures.

### The dynamics of drug-target engagement and epigenomic landscape during EMT

**Fig. 4:**
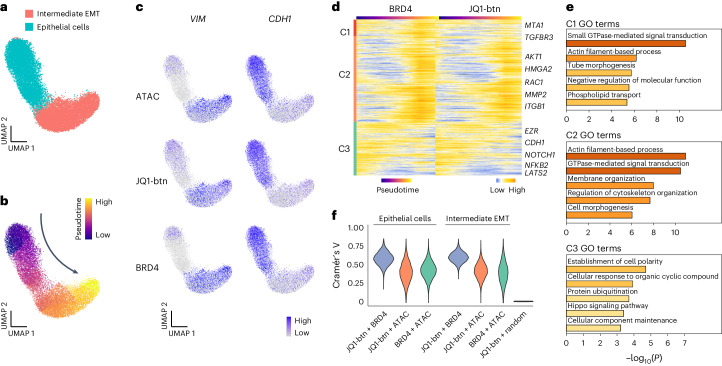
Single-cell EpiChem enables dynamic mapping of JQ1-btn genomic binding and its target BRD4 engagement in human CRC organoids. **a**, UMAP showing scEpiChem (JQ1-btn, BRD4 and ATAC) in human CRC organoids (*n* = 19,983), identified as epithelial cells (*n* = 10,981) and Intermediate EMT cells (*n* = 9,004). **b**, The pseudotime trajectory of the EMT progression. **c**, UMAP projections showing the gene activity scores of *VIM* and *CDH1*. **d**, Heatmaps showing dynamic genomic signals of BRD4 and JQ1 along the pseudotime. Rows were clustered by hierarchical co-clustering and smoothed by the step size of one. Representative genes in each cluster were labeled on the right. **e**, Top five enriched GO terms of each small molecule (C1:1,384, C2:3,615, C3:2,917) are shown on the right. The *P* values of GO term enrichment analysis were calculated using a hypergeometric test, with two-sided statistical tests and adjustments for multiple comparisons employing the Benjamini–Hochberg method. **f**, Violin plots showing Cramér’s V of association between JQ1-btn + BRD4 (median 0.69), JQ1-btn + ATAC (median 0.48), BRD4 + ATAC (median 0.51) in epithelial cells (*n* *=* 10,981); and JQ1-btn + BRD4 (median 0.71), JQ1-btn + ATAC (median 0.50), BRD4 + ATAC (median 0.47) in Intermediate EMT cells (*n* = 9,004) and JQ1-btn + random (*n* = 1,261, median 0.03). The same number of simulated random genomic regions (42,782) as for BRD4 peaks was used. Source data

**Extended Data Fig. 7:**
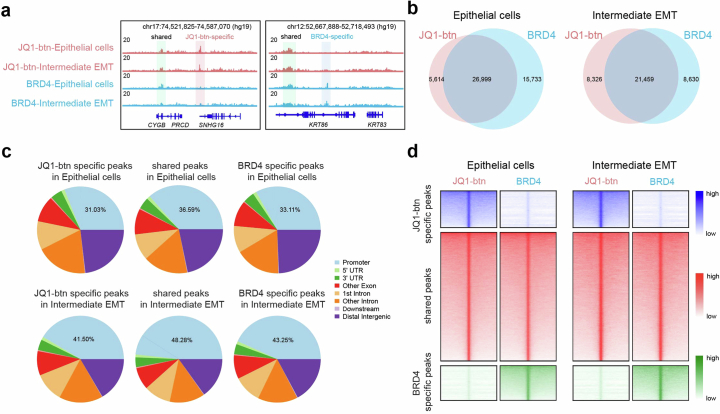
The discrepancies of binding site of JQ1-btn and its target BRD4 were revealed through scEpiChem data in CRC organoids. **a** Track view of JQ1-btn and BRD4 signals on the representative loci in epithelial cells and Intermediate EMT. **b** Venn diagram showing peak overlap of JQ1-btn and BRD4 in epithelial cells and Intermediate EMT. Overlap peak numbers of JQ1-btn and BRD4 in epithelial cells (26,999) and Intermediate EMT (21,459); JQ1-btn specific peak numbers in epithelial cells (5,614) and Intermediate EMT (8,326); BRD4 specific peak numbers in epithelial cells (15,733) and Intermediate EMT (8,630). **c** Genomic distributions of scEpiChem peaks of JQ1-btn and BRD4 in epithelial cells and Intermediate EMT. **d** Heatmap showing JQ1 and BRD4 binding signals of scEpiChem data in JQ1-specific (top), shared (middle) and BRD4-specific (bottom) peaks in epithelial cells and Intermediate EMT.

To further unravel the intricate relationship between drug binding sites and their protein targets in the context of chromatin states, we performed scEpiChem, including ATAC modality with human CRC organoids by simultaneously measuring JQ1-btn, BRD4 and ATAC (Extended Data Fig. 6j). Similarly, as shown in Extended Data Fig. 5, we also assessed the subtle differences between JQ1-btn and its target BRD4 in CRC organoids (Extended Data Fig. 7a–d). As by H3K27ac profiles, clustering single cells by ATAC profiles identified two major populations: epithelial (10,981) and intermediate EMT cells (9,004) (Fig. 4a). We conducted pseudotime analysis on CRC organoid lineages using the ATAC profiles, delineating a hypothetical differentiation trajectory progressing from epithelial to intermediate EMT cells (Fig. 4b). We found strong JQ1-btn BRD4 and ATAC signals at the *VIM* loci in intermediate EMT cells and *CDH1* at the loci in epithelial cells (Fig. 4c). We established 7,916 putative JQ1-to-BRD4 target links associated with EMT (Fig. 4d), and categorized these links into three clusters along the dynamic trajectory of ATAC signals from epithelial to intermediate EMT cells (Fig. 4d). C1 and C2 regions were associated with cooperative increases in JQ1-btn and BRD4 signals (C1, 17.5% of all tested regions; C2, 45.7% of all tested regions). Nevertheless, the signals from C1 JQ1-btn exhibited a modest increase in tandem with the BRD4 signal during the EMT process, whereas the signals from Cluster 2 JQ1-btn showed a cooperative elevation with BRD4. We carried out GO) analysis and found that genes in C1–C2 were strongly enriched for small GTPase-mediated signal transduction and actin filament-based processes (Fig. 4e). C3 links were associated with simultaneous decrease in JQ1-btn and BRD4 signals during EMT (37.8% of all tested regions). Genes in C3 were strongly enriched for Hippo signaling pathway, including Large Tumor Suppressor Kinase 2 gene (*LATS2*). *LATS2* plays a central role in the Hippo pathway and inhibits the EMT process^44–47^. We used Cramér’s V to quantify the degree to which co-enrichment between JQ1-btn and BRD4 existed in the same genes in the same single cells. Intermediate EMT cells (Cramér’s V, 71%) exhibited co-enrichment as much as in epithelial cells (Cramér’s V, 69%) (Fig. 4f). Together, our data shed light on the regulatory interplay of drug genomic binding and epigenomic landscape. These results may provide insights into understanding of drug response heterogeneity.

### Dissecting small-molecule binding profiles reveals differential drug response across cell types in human CRC organoids

**Fig. 5:**
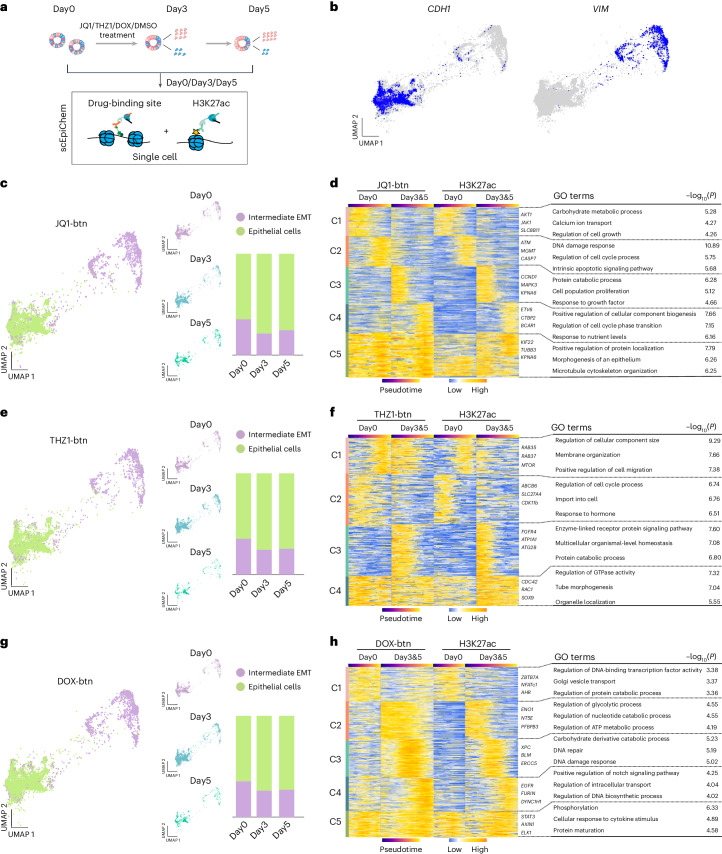
Single-cell EpiChem identifies drug sensitivity across cell types with drug genomic binding sites and chromatin states in human CRC organoids. **a**, Experimental workflow for small-molecule drug treatment of human CRC organoids. **b**, UMAP projections showing the gene activity scores of *VIM*, *CDH1*. **c**, UMAP showing scEpiChem (JQ1-btn and H3K27ac) in human CRC organoids (*n* = 8,797), identified as epithelial cells (*n* = 6,334) and intermediate EMT cells (*n* = 2,463). The stacked bar plot shows the proportion of different cell types at each time point (right). **d**, Heatmaps showing dynamic genomic signals of JQ1-btn along the pseudotime. Cells were ordered by 33,131 peaks in JQ1-btn with 229 columns in Day0 (untreated), 228 columns in Day3&5 (treated). The top three enriched GO terms in each cluster are shown (right). **e**, UMAP showing scEpiChem (THZ1-btn and H3K27ac) in human CRC organoids (*n* = 9,574), identified as epithelial cells (*n* = 6,675) and intermediate EMT cells (*n* = 2,899). **f**, Heatmaps showing dynamic genomic signals of THZ1-btn along the pseudotime. Cells were ordered by 22,054 peaks in THZ1-btn with 171 columns in Day0 (untreated) and 193 columns in Day3&5 (treated). The top three enriched GO terms in each cluster were shown (right). **g**, UMAP showing scEpiChem (Dox-btn and H3K27ac) in human CRC organoids (*n* = 9,239), identified as epithelial cells (*n* = 6,318) and intermediate EMT cells with a high EMT score (*n* = 2,921). **h**, Heatmaps showing dynamic genomic signals of Dox-btn along the pseudotime. Cells were ordered by 17,908 peaks in Dox-btn with 174 columns in Day0 (untreated) and 282 columns in Day3&5 (treated, columns referring to metacells with 50 single cells each). The top three enriched GO terms in each cluster were shown. The top three enriched GO terms in each cluster are shown. *P* values of GO term enrichment analysis in **d**, **f** and **h** were calculated using hypergeometric test, with two-sided statistical tests and adjustments for multiple comparisons employing the Benjamini–Hochberg method. Source data

**Extended Data Fig. 9:**
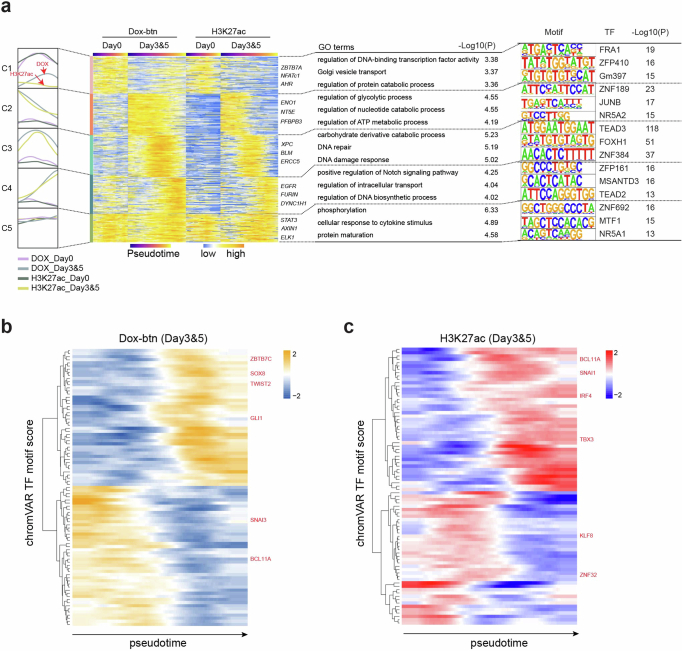
The dynamics of drug engagement and epigenomic landscape during EMT. **a** Heatmaps showing dynamic genomic signals of Dox-btn and H3K27ac along the pseudotime. Left, aggregate curves showing signals of Dox-btn and H3K27ac in Day0 and Day3&5 along the pseudotime, respectively. Middle, heatmaps showing dynamic genomic signals of Dox-btn along the pseudotime. Cells were ordered by 17,908 peaks in Dox-btn with 174 columns in Day0 (untreated) and 282 columns in Day3&5 (treated, columns referring to metacells, 50 single cells each). Rows were clustered by hierarchical co-clustering and smoothed by the step size of one. The binding dynamics of the signals (peaks with less than 10 reads were removed) of JQ1-btn was presented along the EMT progression. Right, Top 3 enriched GO terms in each cluster were shown. De novo TF motifs in peaks were discovered using Homer. P value was calculated by the Binomial test. The results of Gene Ontology (GO) term enrichment analysis using a hypergeometric test, with two-sided statistical tests and adjustments for multiple comparisons employing the Benjamini-Hochberg method. **b-c** ChromVAR identifying TF dynamics during EMT in differential regions between Dox-btn and H3K27ac for Day3&5 CRC samples based on the signals in C1 peaks as in (**a**). Among the dynamic TF genes with increasing TF activity score along pseudotime (44 genes in Dox-btn, 43 genes in H3K27ac, FDR < 0.001), only 7 TFs are shared; Among the dynamic TF genes with decreasing TF activity score along pseudotime (46 genes in Dox-btn, 40 genes in H3K27ac, FDR < 0.001), only 4 TFs are shared.

Understanding the complex molecular mechanisms underlying drug resistance is essential for developing targeted and personalized cancer therapies^48^. We hypothesize that during drug treatment of CRC organoids, the epigenomic landscape within resistant and sensitive cells undergo changes associated with differential small-molecule drug binding, leading to distinct cellular responses to the drugs. To accurately capture the relationship between epigenetic landscape and genomic binding of small molecules in heterogeneous cell populations of CRC organoids before and after drug treatment, we treated organoids with JQ1, THZ1, Dox or dimethylsulfoxide (DMSO) at half-maximum inhibitory concentration (IC_50_) for 5 days (Extended Data Fig. 1c–e). We collected single cells at three time points (day 0 as untreated, day 3 and day 5) and performed simultaneous profiling of small molecules and H3K27ac, capturing a total of 40,682 single cells (Fig. 5a and Extended Data Fig. 6a,b). We utilized the H3K27ac profiles to categorize cells into epithelial cells (33,207) and intermediate EMT cells (7,475) based on associated marker genes (Fig. 5b). To further examine the impact of JQ1, THZ1 and Dox treatments on CRC organoids, we elucidated the proportions of each cell population at three time points during drug treatment. We observed that during the 5-day drug treatment, none of the drugs led to the absence of any cell population (Fig. 5c–h). To further investigate the differential small-molecule binding sites between resistant and sensitive cells, we divided cells into two groups: untreated (day 0 and DMSO) and treated (day 3 and day 5). We selected differential H3K27ac peak regions between untreated and treated cells, overlapping with the peak regions of small molecules. We used *k*-means clustering to cluster these regions (JQ1-btn, 33,131 peaks; THZ1-btn, 22,054 peaks; Dox-btn, 17,908 peaks) based on distinct genomic distributions of small molecules along the dynamic trajectory by H3K27ac signals, spanning from epithelial to intermediate EMT cells. During the EMT process, C1–C2 represented regions where JQ1-btn was bound in drug untreated cells. C3–C4 mainly enriched for the JQ1-btn binding regions in drug-resistant cells, including the regulation of cell cycle phase transition (Fig. 5c,d). For THZ1-btn, the binding sites in drug-resistant cells were mainly enriched in organismal-level homeostasis and the enzyme-linked receptor protein signaling pathway (Fig. 5e,f). In contrast, Dox-btn signals in drug-resistant cells were predominantly enriched in DNA damage response, regulation of nucleotide catabolic processes and positive regulation of the Notch signaling pathway (Fig. 5g,h). We also witnessed a discrepancy between ‘Dox_Day3&5’ and ‘H3K27ac_Day3&5’. Dox-btn binding signals displayed a gradually increasing pattern, whereas H3K27ac signals in C1 peaks decrease continuously along the pseudotime. The discrepancies between Dox-btn and H3K27ac in C1 were related to biological processes, including regulation of DNA-binding transcription factor activity (Extended Data Fig. 9a). Thus, we further used chromVAR^49^ to investigate the transcription factor activity that defines different cell states within subpopulations along pseudotime during EMT. A vast majority of transcription factors along the pseudotime was unique in both groups. Notably, although Dox-btn and H3K27ac shared C1 peaks called from aggregate profiles, the dynamic transcription factor activity was not the same between Dox-btn and H3K27ac for Day3&5 samples when examined at a single-cell level. For example, BCL11A, as an oncogene^50,51^, displayed a decreasing trend by the transcription factor motif score along pseudotime in Dox-btn for Day3&5, whereas the BCL11A transcription factor score continuously increased in H3K27ac for Day3&5 samples. This difference might be related to the discrepancy between Dox-btn binding and H3K27ac signals across pseudotime in C1 (Extended Data Fig. 9b,c). These results indicate the differential subsets of TFs involved in response to Dox-btn treatment as well as associated enhancer regulation during cell state transition. Therefore, we elucidated the differences in small-molecule binding before and after drug treatment, establishing the correlation between the epigenetic context and drug sensitivity.

## Discussion

scEpiChem offers a unique method to jointly measure genomic interactions of small molecules and epigenetic states, including histone modifications and chromatin accessibility in single cells. One of the notable strengths of scEpiChem lies in its ability to capture the heterogeneity of small-molecule drug genomic binding within tumors or tissues, a facet often overlooked in traditional bulk assays. The utilization of split-and-pool barcoding enables the retrieval of hundreds of thousands of single-cell profiles in a single experiment, which is readily scaled up to millions of single cells by increasing more sample barcodes in the first round. The method’s efficacy is demonstrated through successful profiling of three biotinylated inhibitors (JQ1, THZ1 and Dox) along with additional epigenomic features, showcasing its versatility and potential for broad applications. Beyond the overall similarity between drugs and their target proteins, scEpiChem enables the identification of the subtle differences between drug–chromatin binding and its target protein binding (Extended Data Figs. 5 and 7). Therefore, the existence of both shared and unique binding sites of drugs and their known protein targets would also partly justify the utility of scEpiChem for drug profiling, rather than merely profiling target protein-DNA binding to predict the drug binding. Single-cell mapping of drugs targeted to cellular DNA informs cell state-specific targets and regulation of gene program, together with co-assayed multimodal epigenetic landscape, providing mechanistic insights into how drugs act in the cellular and epigenomic context and regulatory interplay within therapeutic modalities. Leveraging the power of scEpiChem data in patient organoids holds great promise to explore the cellular response and resistant mechanisms of anticancer small molecules targeting the genome or epigenome. In the study of drug resistance mechanisms, scEpiChem has offered a comprehensive, high-throughput framework, enabling the capture of cellular heterogeneity of small-molecule response, which is unattainable with traditional bulk assays.

CRC cells undergoing EMT display heightened resistance to therapies, posing a formidable clinical challenge. EMT in CRC correlates with tumor invasion and metastasis, enabling cancer cells to lose cell–cell adhesion and gain increased mobility and invasiveness^39,40,52^. Through the investigation of human CRC organoids, we observed cell type-specific variations in the response to drug treatment. Analyzing small-molecule genomic binding dynamics allowed us to gain a more comprehensive understanding of the cellular transition between drug-resistant and -sensitive states. The capability to simultaneously map small-molecule inhibitors and multiple epigenomic features in single cells opens avenues for studying the diverse interactions between drugs and the dynamic epigenomic landscape. This knowledge is crucial for designing more-targeted and effective therapeutic strategies, such as combination of selected small-molecule drugs for overcoming drug resistance; however, scEpiChem is not without limitations. The reliance on biotinylated small molecules may introduce biases and further efforts are needed to explore alternative labeling strategies.

In conclusion, scEpiChem marks a pioneering step toward unraveling the complexities of small molecule–genome interactions at the single-cell level. Its potential impact spans diverse fields, from drug development to understanding cellular heterogeneity. Addressing current limitations and exploring refinements will be crucial for unlocking the full potential of scEpiChem in advancing our knowledge of drug genomic engagement and paving the way for more-targeted and personalized therapeutic interventions.

## Methods

### Oligonucleotides

The sequences of oligonucleotide primers used in this work are available in Supplementary Table 1.

### Chemical synthesis

The synthesis of Dox-btn was based on the previously reported method by the BioDuro-Sundia company^8^. NMR spectra were recorded on a Bruker 400 MHz Advance III Spectrometer for 1H NMR in DMSO-d6 and analyzed using MestReNova 14.2.3 software. NMR data were reported as follows: chemical shifts in parts per million (ppm) referring to the solvent residual peak, multiplicities (s, singlet; d, doublet; t, triplet; q, quartet; m, multiplet; br, broad) and coupling constant (*J*) values in Hz. LC–MS was performed on an Agilent ESI-MS (Agilent) connected to an Agilent 1260 Infinity system. Dox (T1020, CAS: 25316-40-9), JQ1 (T2110, CAS: 1268524-70-4) and THZ1 (T3664, CAS: 1604810-83-4) were obtained from TargetMol. JQ1-btn (HY-145667, CAS: 1635437-52-3) and THZ1-btn (HY-128867, CAS: 1604811-14-4) were obtained from MedChemExpress^53,54^.

### Human participants

This study was approved by the Research Ethics Committee of the Institute of Basic Medical Sciences, Chinese Academy of Medical Sciences, School of Basic Medicine Peking Union Medical College (2022173). Written informed consent was obtained from each patient. Fresh tumor tissues were collected from treatment-naive patients with CRC who underwent primary curative resection within 30 min after the operation.

### Cell and organoids culture

The mES cells (original from Novus Biologicals, NBP1-41162) were provided by H. Deng, Peking University, and maintained in this laboratory. The HGC27 cell line (original from ECACC, CVCL_1279) and SNU16 cell line (original from ATCC, CRL-5974) were provided by S. Shu, Peking University Cancer Hospital & Institute, and maintained in this laboratory. The human K562 cell line (original from ATCC, CCL-243) was maintained in this laboratory. All cells were cultured at 37 °C with 5% CO_2_. K562 cells were cultured in RPMI 1640 (Gibco) supplemented with 10% fetal bovine serum (FBS; Sigma). SNU16 cells were cultured in DMEM (Hyclone) supplemented with 10% FBS. HGC27 cells were cultured in RPMI 1640 (Gibco) supplemented with 20% FBS. The mES cells were cultured in DMEM (Hyclone) supplemented with 15% FBS, 1% Glutamax (Gibco), 0.1 mM 2-mercaptoethanol (Sigma), 1% MEM nonessential amino acids (Cellgro), 1% nucleoside (Millipore) and 1,000 U ml^−1^ recombinant leukemia inhibitory factor (Millipore). CRC organoids were established as described previously and cultured in CRC organoid medium^55^.

### Cell imaging

SNU16, K562 and HGC27 cells were treated with JQ1 (2 µM), JQ1-btn (2 µM), THZ1 (1 µM), THZ1-btn (1 µM), Dox (3 µM) or Dox-btn (3 µM) for 3 h, respectively. Cells were fixed with 0.25% formaldehyde for 5 min on ice. CRC organoids were treated with JQ1 (5 µM), JQ1-btn (5 µM), THZ1 (5 µM), THZ1-btn (5 µM), Dox (3 µM) or Dox-btn (3 µM) for 6 h. Subsequently, the organoids were fixed with 4% formaldehyde for 10 min. After washing twice with 0.1% BSA/PBS, cells were dropped onto the slides for 2 h at room temperature. Slides were then permeabilized with 0.5% TX-100 in PBS and blocked with 3% BSA/PBS for 30 min. Finally, slides were incubated overnight at 4 °C with primary rabbit monoclonal anti-biotin (CST, 5597S, 1:250 dilution), and goat anti-rabbit Alexa Fluor 488 secondary antibodies (4412S, CST) were used for visualization of drug. Cell nuclei were stained with 4,6-diamidino-2-phenylindole (C1002, Beyotime), and the slides were scanned under a confocal microscope.

### Drug treatment

CRC organoids were cultured in organoid medium supplemented with 5% Matrigel and plated in low-attachment six-well plates. After a 24-h recovery period, the organoids were exposed to either DMSO or small-molecule drugs at their respective IC_50_ concentrations for 3 or 5 days. Following the treatment, the organoids treated with DMSO or small-molecule drugs were collected and subjected to single-cell EpiChem assay for further analysis.

### Cell viability assays

For dose–response assays, drugs were serially diluted in medium. These diluted compounds were then added to 96-well plates, which were initially seeded with 1.5 × 10^3^ cells per well, resulting in a final concentration of 0.1% DMSO. After incubating drug for 96 h, CellTiter-Glo Luminescent Cell Viability Assay (G7572, Promega) was added to each well. The plates were further incubated for 10 min, and luminescence was measured using a SYNERGY H1 microplate reader (BioTek). CRC organoids suspended in 5% Matrigel containing organoid culture medium were plated in 384-well plates at a density of 1,000 per well. Gradient dilutions of drugs were added to the respective wells in the 384-well plates. After 5 days of treatment, the plates were equilibrated to room temperature and cell viability was assessed using the CellTiter-Glo viability assay (Promega), following the instructions provided by the manufacturer.

### Barcoded protein A-Tn5 preparation

The production of PAT was conducted in-house according to the previous protocol^12^. In brief, pET28a-His-pA-Tn5 was transformed into BL21 (DE3)-competent cells. The colonies obtained were then cultured in 500 ml LB medium. To induce PAT expression, 0.2 mM IPTG was added when the optical density of the bacterial culture reached 0.5–0.8. Incubation of the culture was carried out at 23 °C and 80 rpm for 5 h. The bacterial pellets were collected and lysed using HGX buffer, followed by sonication. Genomic DNA in the lysate was precipitated with polyethyleneimine (Sigma). The clear lysate was then applied to a nickel-nitrilotriacetic acid column (QIAGEN) and PAT was eluted from the column using 100 mM imidazole. The concentration of PAT was quantified by Coomassie blue staining after resolution in a 7.5% PAGE gel. Next, PAT was mixed with the annealed barcoded adaptor (Supplementary Table 1) at an equal molar ratio of 37.5 µM, and the mixture was incubated at 25 °C for 1 h. The assembled PAT was either stored at −20 °C or used directly for further experiments.

### Sample fixation

Cell pellets were resuspended in 1 ml 0.1% BSA/PBS, before 7 μl 36.5% formaldehyde was added for incubation on ice for 5 min. To terminate fixation, 14 μl 2.5 M glycine was added and the tubes were inverted several times and kept on ice for 5 min. Subsequently, cells were collected at 300*g* for 3 min at 4 °C and washed with 1 ml 0.1% BSA/PBS twice. Finally, cells were resuspended in cold 1% BSA/PBS and nine volumes of cold methanol were added dropwise on ice for storage at −80 °C.

### EpiChem

#### In vivo

Cells were treated with 10 µM JQ1-btn for 15 min first; then cells were collected and fixed. Chromatin interactions of small molecules were captured by anti-biotin antibodies in antibody buffer (20 mM HEPES, pH 7.5, 150 mM NaCl, 0.5 mM Spermidine (Sigma), 1× Protease Inhibitor Cocktail (Roche), 10 mM sodium butyrate, 0.5 mM EDTA, 0.01% Triton X-100 and 0.01% digitonin) at 4 °C overnight. Then targeted tagmentation and library preparation were undertaken as follows.

#### In vitro

Cells after fixation were incubated with 2.5 µM JQ1-btn with anti-biotin antibodies in antibody buffer at 25 °C for 1 h. After washing in DIG wash buffer twice (20 mM HEPES, pH 7.5, 150 mM NaCl, 0.5 mM Spermidine, 10 mM sodium butyrate and 0.01% digitonin), cells were resuspended in 100 µl antibody buffer with secondary antibodies (Invitrogen, A32790, 1:500 dilution), followed by incubation at 4 °C for 30 min. Cells were washed twice in DIG wash buffer and incubated 9 µg ml^−1^ PAT–MEA and 9 µg ml^−1^ PAT–MEB in DIG-300 wash buffer (20 mM HEPES, pH 7.5, 300 mM NaCl, 0.5 mM Spermidine, 1× Protease Inhibitor Cocktail, 10 mM sodium butyrate, 0.01% Triton X-100 and 0.01% digitonin). Cells were washed twice in DIG-300 wash buffer and resuspended in 50 µl reaction buffer. The reaction was initiated at 37 °C for 1 h and terminated with DIG-300 wash buffer with 5 mM EDTA at room temperature for 5 min. Then, 2,000 cells were washed and resuspended in 4 µl Lysis Buffer (10 mM Tris-HCl, pH 8.5, 0.05 % SDS and 0.1 mg ml^−1^ Proteinase K). Following lysis at 55 °C for 15 min, 1 µl 10 mM phenylmethylsulfonyl fluoride (PMSF) and 1 µl 1.8% Triton X-100 were added to each well and the plate was incubated at 37 °C for 10 min to quench SDS. A total of 50 µl of PCR mix was added to the same tube comprising 25 µl 2× Tag Master mix, 0.5 µl 10 µM Nextera i7 primer, 0.5 µl 10 µM Nextera i5 primer, 1 µl 25 mM MgCl_2_ and 16 µl ddH_2_O. PCR enrichment was then conducted with one cycle of 72 °C for 5 min, one cycle of 95 °C for 2 min and 11 cycles of 98 °C for 30 s, 63 °C for 30 s and 72 °C for 1 min, followed by one cycle of 72 °C for 7 min and a hold at 4 °C. Afterward, 50 µl (0.9×) of custom AMPure XP beads were added to each well, mixed thoroughly, and DNA was purified and eluted using 10 µl ddH2O. For double-size selection purification, 0.5× + 0.5× of AMPure beads were used. DNA was purified and eluted with 20 µl ddH2O.

### Single-cell EpiChem

#### Probe–primary antibody–PAT T7 complex incubation and tagmentation

A small-molecule primary Ab–PAT T7 complex was assembled as previously described^10,28^ with a few modifications. Compound stock solution in DMSO was diluted to 10 µM in probe solution (2 mM EDTA, 0.1% BSA (Sigma) and 0.05% digitonin in wash buffer). Then, 3.34 μl probe solution (10 µM), 0.5 μg antibody (3.33 pmol), 0.22 μl pre-assembled T7-barcoded PAT (8.25 pmol) and 5 μl antibody buffer were mixed thoroughly and incubated at 25 °C for 1 h. Cells were resuspended in 95 µl antibody buffer with the addition of 5 µl of primary Ab–PAT T7 complex and incubated at 4 °C for 4 h (For JQ1-btn, the final concentration was diluted to 2.5 µM and incubated at 25 °C for 1 h. For THZ1-btn, the final concentration was diluted to 0.5 µM and incubated at 4 °C for 4 h. For Dox-btn, the final concentration was diluted to 0.25 µM and incubated at 25 °C for 1 h). Cells were washed three times with DIG-300 wash buffer, and were resuspended in 50 µl reaction buffer consisting of 10 mM TAPS-NaOH (pH 8.3), 5 mM MgCl_2_, 1 mM dithiothreitol, proteinase inhibitor and 10 mM sodium butyrate. The reaction was initiated at 37 °C for 1 h. The reaction was stopped by incubating cells in 180 μl DIG-300 wash buffer with 5 mM EDTA for 5 min.

#### Primary antibody–PAT T7 complex incubation and tagmentation

The 0.5 μg antibody (3.33 pmol), 0.22 μl pre-assembled T7 barcoded PAT (8.25 pmol) and 5 μl wash buffer were mixed thoroughly and incubated at 25 °C for 1 h. After washing three times with DIG-300 wash buffer, cells were resuspended in 95 µl antibody buffer with 5 μl primary Ab–PAT T7 complex and incubated at 25 °C for 1 h. Cells were washed three times with DIG-300 wash buffer and resuspended in 50 µl reaction buffer. The reaction was initiated at 37 °C for 1 h. The reaction was stopped by incubating cells in 180 μl DIG-300 wash buffer with 5 mM EDTA at room temperature for 5 min.

#### Secondary antibody incubation and tagmentation

A secondary antibody diluted at 1:500 was added to 100 µl antibody buffer, followed by incubation at 4 °C for 15 min. After washing twice with DIG-300 wash buffer, cells were resuspended in 100 µl antibody buffer with 9 µg ml^−1^ of barcoded PAT-T5, followed by incubation at 4 °C for 30 min. Cells were washed twice with DIG-300 wash buffer and resuspended in 50 µl reaction buffer. The reaction was initiated at 37 °C for 1 h and terminated with 180 μl DIG-300 wash buffer with 5 mM EDTA for 5 min.

#### Chromatin tagmentation

Chromatin tagmentation was carried out as previously described^56^. Cells were suspended in 50 μl reaction buffer with 2.5 μM Tn5–T5/T7 transposome complex. The mixture was then incubated at 30 °C for 30 min and terminated with 180 μl DIG-300 wash buffer with 5 mM EDTA for 5 min.

#### Hybridizations and ligation

Ligation-based barcoding was carried out as previously described with a few modifications^27^. Cells were washed twice with NSB buffer and resuspended in 4.5 ml Hybridization Mix (1× T4 ligation buffer, 0.05% Triton X-100 and 0.25× NSB). Then, 40 µl cells were added to each of the 96 wells in the first-round barcoding plate, which already contained 10 µl annealed DNA barcodes and was incubated for 30 min at 25 °C (300 rpm). Then, 10 µl of round 1 blocking oligonucleotides was added into the plate and incubated for 30 min at 25 °C (300 rpm). Cells from all 96 wells were combined and 50 µl cells were added to each of the 96 wells in the second-round barcoding plate and incubated for 30 min at 25 °C (300 rpm). Then, 10 µl of round 2 blocking oligonucleotides was added into the plate, and incubated for 30 min at 25 °C (300 rpm). Cells from all wells were combined and washed twice with NSB buffer and centrifuged at 1,000*g* for 3 min. Subsequently, cells were resuspended in 200 µl ligation mix composed of 1× T4 ligation buffer, 20 U µl^−1^ T4 DNA ligase, 0.05% Triton X-100 and 0.2× NSB, and incubated for 30 min at 25 °C (300 rpm).

#### Redistributing cells and releasing DNA

Cells were washed and filtered through a 70-µm cell strainer to remove cell clumps. Subsequently, 1,000–3,000 cells were added into each well of new 96-well plates that contained 4 µl of Lysis Buffer. Following brief lysis at 55 °C for 15 min, 1 µl 10 mM PMSF and 1 µl 1.8% Triton X-100 were added to each well and the plate was incubated at 37 °C for 10 min to quench SDS.

#### Library preparation

A total of 50 µl of PCR mix was added to each well, comprising 10 µl 5× KAPA HiFi buffer, 1 µl 10 mM dNTP Mix, 2 µl 10 µM P7 connector primer, 2 µl 10 µM TruSeq P5 primer, 0.5 µl 1 U µl−1 KAPA HiFi HotStart DNA polymerase, 1 µl 25 mM MgCl_2_ and 26.5 µl ddH_2_O. PCR enrichment was then conducted with one cycle of 72 °C for 5 min, one cycle of 95 °C for 3 min, and 12 cycles of 98 °C for 20 s, 65 °C for 30 s and 72 °C for 30 s, followed by one cycle of 72 °C for 5 min and a hold at 4 °C. Afterward, 0.9× custom AMPure XP beads were used for DNA purification and followed by the addition of 50 µl PCR mix containing 10 µl 5× KAPA HiFi, 1 µl 10 mM dNTP Mix, 2.5 µl 10 µM P7 primer, 2.5 µl 10 µM P5 primer and 0.5 µl 1 U µl^−1^ KAPA HiFi HotStart DNA polymerase. The second PCR enrichment was carried out with one cycle at 72 °C for 5 min, one cycle at 98 °C for 3 min, six cycles at 98 °C for 20 s, 65 °C for 30 s, 72 °C for 1 min, and one cycle at 72 °C for 1 min, followed by hold at 4 °C. Finally, the library was purified once with 0.5× + 0.4× AMPure XP beads.

### Data processing

#### scEpiChem data processing

scEpiChem data were processed to generate unique and non-duplicated reads as previously described^12^. In brief, we evaluated the quality of scEpiChem sequencing data by FastQC (v.0.11.5). Low-quality bases and adaptors were removed by Cutadapt (v.1.11) with the following parameters: -q 20 -O 10–trim-n -m 30–max-n 0.1. Clean paired-end EpiChem reads were then mapped to the human and mouse reference genome hg19 and mm10 using Bowtie2 (v.2.2.9)^57^. The mapped reads with MAPQ greater than 30 were considered as uniquely mapped reads, which were sorted using SAMtools (v.1.9) and used for subsequent analyses. PCR duplicates were removed by Picard (v.2.2.4) (http://broadinstitute.github.io/picard) with default parameters. Only uniquely mapped, non-duplicated reads were used for peak calling by MACS2 (v.2.1.1)^58^ and the following analyses.

#### Visualization and correlation analysis of scEpiChem data

We used the bamCoverage function in deepTools (v.2.2.3)^59^ to calculate genome coverage in BAM files and generated track files (bigWig format) for scEpiChem data. For the visualization of the signals of histone modifications/drug binding at specific loci, bigwig files were uploaded into Integrative Genomics Viewer (v.2.3)^60^ together with corresponding reference tracks. To evaluate correlations of JQ1-btn and BRD4 in K562 and HGC27, we calculated the normalized average scores in Chem-map BRD4 peak regions (51,963 peaks)^10^. The correlation was calculated between different groups and plotted by deepTools (v.2.2.3).

#### Collision rate

To evaluate the efficiency of split-and-pool strategy for single-cell labeling of scEpiChem, we generated scatter-plots using the proportion of reads mapped to human or mouse genome in each barcode combination in Fig. 1. Barcodes with <95% of aligned reads mapped to one species were classified as collisions^61^.

#### Single-cell scEpiChem data processing

The scEpiChem data were demultiplexed by custom scripts. In brief, the analysis pipeline of scEpiChem data processing consisted of following steps: (1) creating the single-cell whitelist using UMI-tools (v.1.1.2)^62^ based on the molecular design of scEpiChem with the parameter ‘–set-cell-number=n’; (2) extracting the paired-end reads based on the single-cell whitelist; (3) mapping to the human or mouse reference genomes by bowtie2 (v.2.2.9); (4) keeping unique mapped reads and removing PCR duplicates; and (5) adding cell barcode information to the BAM files and generating single-cell BAM files^63^.

#### Single-cell quality control and filtering

After removing doublets or multiplets in each batch by DoubletFinder (doubletFinder_v.3)^64^, we retained ~85% single cells (>400 unique fragments) with tri-omics information subjected to downstream analysis.

#### FRiP score

For each single-cell BAM file, the FRiP score calculation proceeds as follows: conversion of BAM to BED format using bedtools bamtobed, followed by intersection of reads with the peak regions using bedtools intersect. This tool is employed to count the number of reads that overlap with any of the defined peak regions, using the BED format reads and the peak file as input. Calculation of total read count within each BAM file was performed using SAMtools view -c. The FRiP score for each cell was then calculated as the ratio of reads intersecting with peaks to the total number of reads, expressed as a decimal to four significant figures. The computed FRiP scores for all analyzed cells were collected and written to a text file, preserving both the cell identifier and the corresponding FRiP score.

#### Dimensionality reduction and visualization of tri-omics in single cells

Single-cell data were processed using cisTopic v.3 (ref. ^65^) to generate a cell-peak matrix, and further processed using Signac (v.1.9.0)^66^ to perform term frequency-inverse document frequency (TF-IDF) normalization and latent semantic indexing clustering. The gene ACTIVITY assay in Seurat Object of cell-peak or cell-bin matrix for each small molecular and histone modification was generated by GeneActivity function of Seurat v.4 (ref. ^38^) with default parameters. The Seurat Object of AVTIVITY assay was processed using principal-component analysis based on the top 2,000 highly variable genes by the ‘FindVariableGenes’ function in Seurat. Cell clusters on UMAP were identified using the ‘FindClusters’ function of Seurat.

#### Pseudotime analysis

We conducted pseudotime analysis using Monocle3 (ref. ^67^). Highly variable genes identified by ‘FindVariableGenes’ function of Seurat were used to estimate similarities between cells. Pseudotimes were calculated according to similarities in the transcriptome or epigenome to reflect cell relationships during differentiation. Further, cells were ordered by pseudotime as shown in Figs. 2d and 3d.

#### Genomic annotation analysis

Genes near to regulatory elements were selected with the annotatePeaks.pl function in Homer^68^. GO analysis for biological processes was performed using Metascape (http://metascape.org)^69^, with Benjamini–Hochberg *P* value correction^70^.

#### Data downsampling

The sampling rates were set at predetermined values, including 80% to 20%. Each sampling rate corresponded to an independent downsampling process. Utilizing the SAMtools view command (with -s specifying the sampling rate and -b for generating BAM format files), a proportionate subset of reads was randomly selected from the original BAM files to create new BAM files for each sampling rate.

#### Cluster error rate calculation

A cisTopic object was created from the downsampled BAM files and genomic region files in BED format using the createcisTopicObjectFromBAM function. Collapsed Gibbs Sampling models were then executed to explore the optimal model selection among cistopics. Model selection was assessed based on the log-likelihood iteration plot. For the finalized model, dimensionality reduction was conducted using the UMAP method, which subsequently formed the basis for clustering analysis of cells. Hierarchical clustering was performed using the fastcluster package, categorizing cells into two groups based on their UMAP coordinates. This error rate was defined as the proportion of cells incorrectly grouped relative to the total number of cells. Specifically, clustering outcomes were compared against known cell types, with any incongruent allocations considered erroneous.

#### Statistics and reproducibility

All EpiChem experiments were independently performed at least twice. The Seurat package implemented in R was used to identify variable genes by calculating the variance and finding the differentially expressed. The Monocle 3 package was used to calculate pseudotime as described above. Other statistical analyses for EpiChem were mainly performed between groups of peaks or segments of the genome. The statistical tests are indicated in the figure legends.

### Reporting summary

Further information on research design is available in the Nature Portfolio Reporting Summary linked to this article.

## Online content

Any methods, additional references, Nature Portfolio reporting summaries, source data, extended data, supplementary information, acknowledgements, peer review information; details of author contributions and competing interests; and statements of data and code availability are available at 10.1038/s41592-024-02360-0.

## Supplementary information

Supplementary Information Supplementary Protocols.

Reporting Summary

Supplementary Table 1 The sequences of custom primers used in this work.

Supplementary Table 2 Pairwise comparison between EpiChem and Chem-map in bulk assay.

Supplementary Table 3 The data quality of EpiChem and scEpiChem data.

## Source data

Source Data Fig. 1 Statistical source data.

Source Data Fig. 2 Statistical source data.

Source Data Fig. 3 Statistical source data.

Source Data Fig. 4 Statistical source data.

Source Data Fig. 5 Statistical source data.

## Figures and Tables

**Extended Data Fig. 8:**
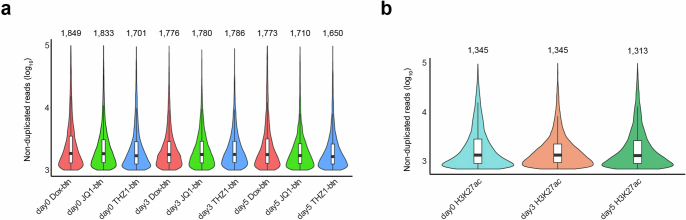
Characterization of scEpiChem data in human CRC organoids after treatment with invidual small molecules. **a** Violin plot showing the median non-duplicated reads per cell of Dox-btn (n = 9,239), JQ1-btn (n = 8,797) and THZ1-btn (n = 9,574) in day0, day3 and day5 of after removal of single cell with less than 1,000 raw reads. Numbers on the top of violin plot indicate the median value. Boxes in the violin plots: center marks the median; edges of boxes define the 25th and 75th percentiles. **b** Violin plot showing the median non-duplicated reads per cell of H3K27ac in day0 (n = 11,480), day3 (n = 9,678) and day5 (n = 6,452). Numbers on the top of violin plot indicate the median value. Boxes in the violin plots: center marks the median; edges of boxes define the 25th and 75th percentiles.

## Data Availability

The raw sequence data reported in this paper have been deposited in the Genome Sequence Archive (GSA) in the National Genomics Data Center^71,72^, China National Center for Bioinformation/Beijing Institute of Genomics, Chinese Academy of Sciences (accession nos. GSA-Human: HRA005220, HRA005219, https://ngdc.cncb.ac.cn/gsa-human/; GSA: CRA012132, https://ngdc.cncb.ac.cn/gsa/). Other public datasets used in this study were downloaded from the NCBI Gene Expression Omnibus with accession nos. as follows: Chem-map (GSE209713); ENCODE H3K27me3 (GSE31755); and ENCODE ATAC (GSE90409). The GRCh37 reference genome was downloaded from NCBI datasets (https://www.ncbi.nlm.nih.gov/datasets/genome/GCF_000001405.13/). The GRCm38 reference genome was downloaded from NCBI datasets (https://www.ncbi.nlm.nih.gov/datasets/genome/GCF_000001635.20/). Source data are provided with this paper.

## Code availability

Custom scripts used in this study are available from Zenodo at 10.5281/zenodo.11235132 (ref. ^73^).
