# Supplementary material for: Single-cell EpiChem jointly measures drug–chromatin binding and multimodal epigenome
Source: Nat Methods. 2024 Jul 18;21(9):1624–33. doi: 10.1038/s41592-024-02360-0 (PMC11399096; doi:10.1038/s41592-024-02360-0)
Supplement: Supplementary file 1 — Supplementary Protocols. [file 41592_2024_2360_MOESM1_ESM.pdf]

nature methods

Supplementary information https://doi.org/10.1038/s41592-024-02360-0

Single-cell EpiChem jointly measures drug–chromatin binding and multimodal epigenome

In the format provided by the authors and unedited

Supplementary Protocol – Protocol on scEpiChem for jointly measuring drug-chromatin binding and multimodal epigenome

Notes: This protocol for simultaneously profiling drug- and chromatin proteins-DNA interactions with optional chromatin accessibility in the same single cells with ultra-high throughput.

Reagents:

STE buffer: 10 mM Tris-HCl pH 8.0, 50 mM NaCl, and 1 mM EDTA.

10× T4 ligase buffer: (NEB cat# B0202S) Dilute 10× T4 ligase buffer 1:5 with water to create a 2× T4 ligase buffer.

Triton X-100: (VWR Life Science cat# M143) TritonX-100 is supplied at 10%.

Digitonin: (Sigma cat# D141) digitonin is supplied at 5%.

EDTA: (VWR Life Science cat# 0105) EDTA is supplied at 0.5 M.

Glcyine: (VWR Life Science cat# 0167) Glcyine is supplied at 2.5 M.

HEPES: (VWR Life Science cat# 0511) HEPES is supplied at 1 M (pH 7.5).

Sodium butyrate: (Sigma cat# 303410) Sodium butyrate is supplied at 1 M.

Sodium chloride: (Sigma cat# 793566) Sodium chloride is supplied at 5 M.

Sodium Dodecyl SμLafte: (VWR Life Science cat# 0227) SDS is supplied at 10%.

TAPS-NaOH: (Sigma cat# T5316) TAPS-NaOH is supplied at 1 M (pH 8.3).

Methanol: Sigma cat# 494437.

Buffers:

Wash buffer

Reagents	Final concentration	Volume for 50 ml
1 M HEPES pH 7.5	20 mM	1 ml
5 M NaCl	150 mM	1.5 ml
2 M spermidine	0.5 μM	12.5 μl
Sterile water	NA	up to 50 ml

High salt wash buffer

Reagents	Final concentration	Volume for 50 ml

1 M HEPES pH 7.5	20 mM	1 ml
5 M NaCl	300 mM	3 ml
2 M spermidine	0.5 µM	12.5 µl
Sterile water	NA	up to 50 ml

STE buffer

Reagents	Final concentration	Volume for 50 ml
1 M Tris-HCl pH 8.0	10 mM	500 µl
5 M NaCl	50 mM	500 µl
500 mM EDTA	1 mM	100 µl
Sterile water	NA	up to 50 ml

NSB buffer

Reagents	Final concentration	Volume for 50 ml
1 M Tris-HCl pH 7.5	10 mM	500 µl
5 M NaCl	10 mM	100 µl
5% TX-100	0.1%	100 µl
1 M $MgCl_2$	3 mM	150 µl
10% BSA/PBS	0.1%	500 µl
Sterile water	NA	up to 50 ml

Antibody buffer

Reagents	Final concentration	Volume for 1 ml
50 mM EDTA	2 mM	40 µl
10% BSA/PBS	0.1%	10 µl
5% digitonin	0.05%	10 µl
100× protease inhibitor cocktail	1×	10 µl
Wash buffer	NA	up to 1 ml

DIG wash buffer

Reagents	Final concentration	Volume for 10 ml
5% digitonin	0.01%	20 µl

1M sodium butyrate	10 mM	100 µl
Wash buffer	NA	up to 10 ml

DIG-300 wash buffer

Reagents	Final concentration	Volume for 10 ml
5% digitonin	0.01%	20 µl
1M sodium butyrate	10 mM	100 µl
High salt wash buffer	NA	up to 10 ml

Prior to Transposition:

Before conducting formal experiments, it is necessary to prepare 96 round 1 adaptors and 96 round 2 adaptors for two rounds of hybridization.

Prepare the oligonucleotides listed below for two-rounds hybridization:

1. Prepare the stocking solution:

   a. Dissolve the round 1 linker oligos with STE buffer to 100 µM.

   b. Dissolve the round 1 barcode oligos with STE buffer to 100 µM.

   c. Dissolve the round 2 linker oligos with STE buffer to 100 µM.

   d. Dissolve the round 2 barcode oligos with STE buffer to 100 µM.

   e. Dissolve the round 1 blocking oligos with 2× T4 ligase buffer to 100 µM.

   f. Dissolve the round 2 blocking oligos with 0.05% TritonX-100 to 100 µM.

* Dissolve all the DNA oligos to 100 µM, and all the oligos are thawed to room temperature before using.

2. Prepare the working solution:

   a. Working concentration of round 1 adaptors:

Reagents	Final concentration	Volume for 100 µl
100 µM round 1 linker oligos	9 µM	9 µl
100 µM round 1 barcode oligos	10 µM	10 µl
STE buffer	NA	81 µl

   b. Working concentration of round 2 adaptors:

Reagents	Final concentration	Volume for 100 µL
100 µM round 2 linker oligos	11 µM	11 µl
100 µM round 2 barcode oligos	12 µM	12 µl
STE buffer	NA	77 µL

c. Round 1 adaptors and round 2 adaptors annealing:

Temperature	Cycling conditions
95ºC	2 minutes
	-1ºC/cycle, 1 minutes per cycle
20ºC	2 minutes
4ºC	hold

d. Working concentration of round 1 blocking oligos:

Reagents	Final Concentration	Volume for 100 µl
100 µM round 1 blocking oligos	22 µM	220 µl
2× T4 ligase buffer (NEB)	NA	780 µl

e. Working concentration of round 2 blocking oligos:

Reagents	Final Concentration	Volume for 100 µl
100 µM round 2 blocking oligos	26.4 µM	264 µl
0.05% TritonX-100	NA	736 µl

* The plates can be stored at 4ºC for three months.

Sample preparation:

1. Harvest 0.2-1 million cells and digest cells into single-cell suspension.

2. Wash cells by 1 ml 0.1% BSA/PBS twice.

*Wash tubes with 1% BSA/PBS prior to use and use low-retention tubes if possible. This operation can prevent cell/beads loss.

3. Cell pellets are resuspended with 1 ml 0.1% BSA/PBS.

4. Add 7 µl 36.5% FA and incubate the tubes on ice for 5 min.

5. To stop fixation, add 14 µl 2.5M Glycine, invert the tubes several times and keep on ice for 5 min.

6. Collect at 600 g for 3 min at 4 °C and wash cell pellets with 1 ml 0.1% BSA/PBS twice.

7. The cell pellets are resuspended with 100 µl 0.1% BSA/PBS.

8. Fix cells by adding -20°C methanol drop by drop to a final concentration of 90%.

* The prepared sample can be stored at -80°C for at least six months.

Probe-1st Ab-PAT T7 complex incubation:

9. Compound stock solution in DMSO can be diluted to 10 µM in antibody buffer.

10. 3.34 µl compound solution (10 µM), 0.5 µg anti-biotin antibody (3.33 pmol), 0.22 µl pre-assembled T7 barcoded PAT (8.25 pmol), and wash buffer (total 5 µl) are mixed thoroughly and incubated at 25°C for 1 h.

11. Keep cells on ice for 30 min.

12. Collect cells at 1,000 g for 3 min at 4 °C.

13. Wash cells with 1 ml 0.1% BSA/PBS twice.

14. Resuspend cells with 95 µl antibody buffer with 300 mM NaCl and 5 µl probe-1st Ab-PAT T7 complex in one PCR tube, and incubate the reaction at 4°C for 4 h.

Targeted tagmentation:

15. Wash cells three times with 180 µl DIG-300 wash buffer on a tube rotator and incubate at 4°C for 5 min.

16. Centrifuge cells at 300 g for 3 min and discard the supernatant.

17. Resuspend cells with 50 µl cold reaction buffer (10 mM $MgCl_2$, 10 mM TAPS-NaOH pH 8.3, 0.01% digitonin, 1× protease inhibitor cocktail, 10 mM sodium butyrate).

18. Incubate the tubes at 37°C for 1 h.

*The reaction is gently mixed once after 20-min incubation.

19. Add 180 µl DIG-300 wash buffer (0.01% Triton X-100 and 5 mM EDTA) to each tube, mix well, and rotate for 10 min.

20. Wash cells three times with 180 µl DIG-300 wash buffer.

21. Centrifuge cells at 300 g for 3 min and discard the supernatant.

Second 1st Ab-PAT T7 incubation and tagmentation:

22. 0.5 µg antibody (e.g. antibodies against histone modifications or chromatin binding proteins) (3.33 pmol), 0.22 µl pre-assembled T7 barcoded PAT (8.25 pmol), and wash buffer (total 5 µl) are mixed thoroughly and incubated at 25°C for 1 h. Resuspend cells with 95 µl antibody buffer with 300 mM NaCl and 5 µl 1st Ab-PAT T7 complex in one PCR tube, and incubate the system at 25°C for 1 h.

23. Repeat steps 15-21 to achieve the second tagmentation.

Binding 2nd antibody:

24. Resuspend cells with 100 µl cold antibody buffer premixed with 2nd antibody in a 1:500 dilution.

25. Place the tubes on a rotator at 4°C for 30 min.

26. Wash cells twice with 180 µl DIG wash buffer and discard the supernatant.

Binding PAT-T5 and targeted tagmentation:

27. Add 100 µl DIG-300 wash buffer (0.01% Triton X-100, 1× protease inhibitor cocktail, and 10 mM sodium butyrate) containing sample-barcoded 9 µg/ml pre-assembled T5 PAT.

* Different samples are differentiated with different pre-assembled T5 barcoded PAT.

28. Place the tubes on a tube rotator and incubate at 4°C for 1 h.

29. Centrifuge at 300 g for 3 min.

30. Wash cells twice by 180 µl DIG-300 wash buffer (0.01% Triton X-100) on a tube rotator and incubate at 4°C for 5 min.

31. Resuspend cells with 50 µl cold reaction buffer.

32. Incubate the plate at 37°C for 1 h.

*The reaction is gently mixed once after 20-min incubation.

33. Add 180 µl DIG-300 wash buffer (0.01% Triton X-100 and 5 mM EDTA) to each PCR tube, mix well, and rotate for 10 min.

34. Wash cells three times with 180 µl DIG-300 wash buffer (0.01% Triton X-100).

35. Centrifuge the cells at 300 g for 3 min and discard the supernatant.

(Optional) Chromatin accessibility:

36. Cells are suspended in 50 µl of tagmentation mix, which consists of 33 mM Tris-acetate (pH 7.8), 66 mM potassium acetate, 10 mM magnesium acetate, 16% dimethylformamide, 0.01% digitonin, and 2.5 µM of Tn5-S5/S7 transposome complex.

37. The mixture is incubated at 30 °C for 30 min.

38. To stop the reaction, an equal volume of tagmentation stop buffer (containing 10 mM Tris-HCl pH 7.8, 20 mM EDTA pH 8.0, and 2% BSA) is added.

Two round hybridizations:

39. 150 µl NSB buffer is added to each PCR tube and cells are centrifuged at 300 g for 3 min to remove the supernatant.

40. Cells are washed with 180 µl NSB buffer for three times.

41. Cells are mixed and resuspended in 5 ml of hybridization mix (1 × T4 ligase buffer, 0.01 % Triton X-100, and 0.25 × NSB buffer).

42. Cells in hybridization mix (40 µl) are added to each of the 96 wells in the 1st round

barcoding plate, in which each well already contain 10 μl round 1 adaptor.

43. The round 1 barcoding plate is incubated for 30 min at room temperature with gentle shaking (300 rpm) to allow hybridization to occur before adding blocking oligos.

44. 10 μl of round 1 blocking oligos is added followed by incubation for 30 min at 25℃ with gentle shaking (300 rpm).

45. Cells from all 96 wells are combined and redistributed.

46. Cells in hybridization mix (50 μl) are added to each of the 96 wells in the 2nd round barcoding plate, in which each well already contains 10 μl round 2 adaptors (total 60 μl).

47. The round 2 barcoding plate is incubated for 30 min at room temperature with gentle shaking (300 rpm) to allow hybridization to occur before adding blocking oligos.

48. 10 μl of round 2 blocking oligos is added followed by incubation for 30 min at 25℃ with gentle shaking (300 rpm).

49. After adding the round 2 blocking oligos, cells from all wells are combined and centrifuged at 1,000 g for 3 min to remove the supernatant.

Ligation:

50. Cells are washed twice with 1 ml NSB buffer, and centrifuged at 600 g for 3 min.

51. Cells are resuspended in the 200 μl ligation mix (1x T4 ligation buffer, 20U/μl T4 DNA ligase (M0202L, NEB), and 0.05% Triton X-100 and 0.2 × NIB buffer) and incubated for 30 min at 25℃ with gentle shaking (300 rpm).

Redistributing cells and Releasing DNA:

52. Wash cells twice with cell suspension buffer (3 mM $MgCl_2$, 0.01% Triton X-100 and 0.1% BSA/PBS).

53. Cells are filtered through cell strainer to remove cell clumps.

54. Count the cell number and resuspend the cells at the concentration of 1,000-5,000

cells/μl by 0.1% BSA PBS.

55. 1 μl of cells are sorted into each well of a new 96-well plates, containing 4 μl lysis buffer (10 mM Tris-HCl pH 8.5, 0.05 % SDS and 0.1 mg/ml proteinase K) in each well).

56. Incubate the plate at 55°C for 15 min.

57. Add 1 μl 10 mM PMSF and 1 μl 1.8% Triton X-100 to each well and incubate the plate at 37 °C for 10 min to quench SDS.

Amplification of transposed fragment:

58. A total of 50 μl of PCR mix is added to each well, comprising 10 μl 5× KAPA HiFi buffer, 1 μl 10 mM dNTP Mix, 2 μl 10 μM P7 connector primer, 2 μl 10 μM TruSeq P5 primer, 0.5 μl 1 U/μl KAPA HiFi HotStart DNA polymerase, 1 μl 25 mM $MgCl_2$, and 26.5 μl ddH_2O.

59. PCR enrichment is conducted with one cycle of 72 °C for 5 min, one cycle of 95 °C for 3 min, and twelve cycles of 98 °C for 20 s, 65 °C for 30 s, and 72 °C for 30 s, followed by one cycle of 72 °C for 5 min and a hold at 4°C.

60. 45 μl (0.9×) of custom AMPure XP beads are added to each well and mixed thoroughly, and DNA is purified and eluted in 10 μl of ddH_2O.

61. For double size selection purification, 0.5× + 0.4× of AMPure beads are used.

62. DNA is purified and eluted with 25 μl ddH_2O, followed by addition of 25 μl of PCR mix containing 10 μl 5× KAPA HiFi, 1 μl 10 mM dNTP Mix, 2.5 μl 10 μM P7 primer, 2.5 μl 10 μM P5 primer, and 0.5 μl 1 U/μl KAPA HiFi HotStart DNA polymerase, 1 μl 25 mM $MgCl_2$ and ddH_2O.

63. The second PCR enrichment is carried out with one cycle at 72 °C for 5 min, one cycle at 98 °C for 3 min, six cycles at 98 °C for 20 s, 65 °C for 30 s, 72 °C for 1 min, and one cycle at 72 °C for 1 min, followed by hold at 4 °C.

64. Finally, the library is purified once with 0.9× AMPure XP beads, and the DNA part

library is obtained after purification and elution with ddH_2O.
