# Supplementary material for: Single-cell EpiChem jointly measures drug–chromatin binding and multimodal epigenome
Source: Nat Methods. 2024 Jul 18;21(9):1624–33. doi: 10.1038/s41592-024-02360-0 (PMC11399096; doi:10.1038/s41592-024-02360-0)
Supplement: Supplementary file 2 — Reporting Summary [file 41592_2024_2360_MOESM2_ESM.pdf]

nature portfolio

Corresponding author(s): Aibin He

Last updated by author(s): May 22, 2024

Reporting Summary

Nature Portfolio wishes to improve the reproducibility of the work that we publish. This form provides structure for consistency and transparency in reporting. For further information on Nature Portfolio policies, see our Editorial Policies and the Editorial Policy Checklist.

Statistics

For all statistical analyses, confirm that the following items are present in the figure legend, table legend, main text, or Methods section.

n/a	Confirmed	
☐	☒	The exact sample size (*n*) for each experimental group/condition, given as a discrete number and unit of measurement
☐	☒	A statement on whether measurements were taken from distinct samples or whether the same sample was measured repeatedly
☐	☒	The statistical test(s) used AND whether they are one- or two-sided<br>*Only common tests should be described solely by name; describe more complex techniques in the Methods section.*
☐	☒	A description of all covariates tested
☐	☒	A description of any assumptions or corrections, such as tests of normality and adjustment for multiple comparisons
☐	☒	A full description of the statistical parameters including central tendency (e.g. means) or other basic estimates (e.g. regression coefficient) AND variation (e.g. standard deviation) or associated estimates of uncertainty (e.g. confidence intervals)
☐	☒	For null hypothesis testing, the test statistic (e.g. *F*, *t*, *r*) with confidence intervals, effect sizes, degrees of freedom and *P* value noted<br>*Give P values as exact values whenever suitable.*
☐	☒	For Bayesian analysis, information on the choice of priors and Markov chain Monte Carlo settings
☐	☒	For hierarchical and complex designs, identification of the appropriate level for tests and full reporting of outcomes
☐	☒	Estimates of effect sizes (e.g. Cohen's *d*, Pearson's *r*), indicating how they were calculated

Our web collection on statistics for biologists contains articles on many of the points above.

Software and code

Policy information about availability of computer code

Data collection	All software used in this study has been described in published literature. There are details in the online Methods. Software version: FastQC (v 0.11.5), Bowtie2 (v 2.2.9), Samtools (v 1.9), Picard (v 2.2.4), MACS2 (v 2.1.1), Deeptools (v 2.2.3), IGV (v 2.3), UMI-tools (v 1.1.2), DoubletFinder (doubletFinder v 3), Signac (v 1.9.0), Seurat (v4), Seurat (v5), cisTopic (v 0.3.0), Metascape (http://metascape.org). Further data analysis and presentation are performed with R (v4.2.2).
Data analysis	Custom scripts used in this study are available from https://github.com/Helab-bioinformatics/scEpiChem.<br>All parameters used to analyze data are described in methods section and custom scripts are available upon request. Bowtie2 (v 2.2.9) is used for mapping. Samtools (v 1.9) is used for sorting, selecting uniquely mapping reads and deduplication. Deeptools bamCoverage (v 3.5.1) is used to generate bigwig files. Deeptools (v 2.2.3) plotHeatmap is used for heatmap plotting. Gene ontology terms is performed by Metascape (http://metascape.org). Seurat (v4) and Seurat (v5) is used for scEpiChem analysis.

For manuscripts utilizing custom algorithms or software that are central to the research but not yet described in published literature, software must be made available to editors and reviewers. We strongly encourage code deposition in a community repository (e.g. GitHub). See the Nature Portfolio guidelines for submitting code & software for further information.

Data

Policy information about availability of data

All manuscripts must include a data availability statement. This statement should provide the following information, where applicable:

- Accession codes, unique identifiers, or web links for publicly available datasets
- A description of any restrictions on data availability
- For clinical datasets or third party data, please ensure that the statement adheres to our policy

The raw sequence data reported in this paper have been deposited in the Genome Sequence Archive in National Genomics Data Center53, 54, China National Center for Bioinformation/Beijing Institute of Genomics, Chinese Academy of Sciences (accession numbers: GSA-Human: HRA005220, HRA005219 and GSA: CRA012132, https://ngdc.cncb.ac.cn/gsa/). Other public datasets used in this study were downloaded from NCBI GEO with accession numbers as follows: Chem-map (GSE209713). ENCODE H3K27me3 were downloaded from GSE31755. ENCODE ATAC were downloaded from GSE90409. The GRCh37 reference genome were downloaded from NCBI datasets (https://www.ncbi.nlm.nih.gov/datasets/genome/GCF_000001405.13/). The GRCm38 reference genome were downloaded from NCBI datasets (https://www.ncbi.nlm.nih.gov/datasets/genome/GCF_000001635.20/)

Research involving human participants, their data, or biological material

Policy information about studies with human participants or human data. See also policy information about sex, gender (identity/presentation), and sexual orientation and race, ethnicity and racism.

Reporting on sex and gender	We analysed specimens derived from two male patients.
Reporting on race, ethnicity, or other socially relevant groupings	All patients are from China.
Population characteristics	The specimens of P20 were obtained from a 76-year-old male patient from China with colorectal cancer. The histological type was mucinous adenocarcinoma, and the TNM classification was T4aN0M0. Patient P20 exhibits distinct mutations in different genes, such as TAS1R3 (c.G1486A), FAAP20 (c.G167A) and NPHP4 (c.G1049A). The specimens of P1201 were obtained from a 70-year-old male patient from China with colorectal cancer. The histological type was mucinous adenocarcinoma, and the TNM classification was T4aN2. Patient P1201 exhibits distinct mutations in different genes, such as CDK11B (p.H97R), FBXO44 (p.R129W) and EPHB2 (p.F1040Lfs*43).
Recruitment	Samples were obtained from patients who sought consultation or were being treated at Peking University First Hospital in Beijing. Two colorectal cancer patients were enrolled, and these participants were recruited randomly.
Ethics oversight	The institution involved in the ethical approval of this study is the Institute of Basic Medical Sciences, Chinese Academy of Medical Sciences. The study was reviewed and approved by the Ethics Committee of the Institute of Basic Medical Sciences, Chinese Academy of Medical Sciences (Approval No. 2022173).

Note that full information on the approval of the study protocol must also be provided in the manuscript.

Field-specific reporting

Please select the one below that is the best fit for your research. If you are not sure, read the appropriate sections before making your selection.

☒ Life sciences ☐ Behavioural & social sciences ☐ Ecological, evolutionary & environmental sciences

For a reference copy of the document with all sections, see nature.com/documents/nr-reporting-summary-flat.pdf

Life sciences study design

All studies must disclose on these points even when the disclosure is negative.

Sample size	For cell lines, a total of 17,436 cells were used for capturing multi-omics data with scEpiChem. For organoids, a total of 74,694 cells were used for capturing data from 2 colorectal cancer patients. Since the samples had enough biological material, duplicate data could be generated to confirm the total intracellular findings. This enabled the comparison of various drug-treated and untreated control cells within each patient's sample, taking into account the context of cell identity. No sample szie caculations were performed.
Data exclusions	No data were excluded from the analysis.
Replication	Two or three biological replicates for multiomics libraries are sufficient for assessing the data quality. The scEpiChem protocol was tested on multiple cell lines, including K562, HGC27, and mESC, to ensure its reproducibility across different biological contexts. Additionally, the protocol was applied to colorectal cancer (CRC) organoids and evaluated for its reproducibility to simultaneously capture different omics data.
Randomization	The cells used for each groups were randomly divided at the beginning.

Blinding	We were blinded to group allocation.

Behavioural & social sciences study design

All studies must disclose on these points even when the disclosure is negative.

Study description	*Briefly describe the study type including whether data are quantitative, qualitative, or mixed-methods (e.g. qualitative cross-sectional, quantitative experimental, mixed-methods case study).*
Research sample	*State the research sample (e.g. Harvard university undergraduates, villagers in rural India) and provide relevant demographic information (e.g. age, sex) and indicate whether the sample is representative. Provide a rationale for the study sample chosen. For studies involving existing datasets, please describe the dataset and source.*
Sampling strategy	*Describe the sampling procedure (e.g. random, snowball, stratified, convenience). Describe the statistical methods that were used to predetermine sample size OR if no sample-size calculation was performed, describe how sample sizes were chosen and provide a rationale for why these sample sizes are sufficient. For qualitative data, please indicate whether data saturation was considered, and what criteria were used to decide that no further sampling was needed.*
Data collection	*Provide details about the data collection procedure, including the instruments or devices used to record the data (e.g. pen and paper, computer, eye tracker, video or audio equipment) whether anyone was present besides the participant(s) and the researcher, and whether the researcher was blind to experimental condition and/or the study hypothesis during data collection.*
Timing	*Indicate the start and stop dates of data collection. If there is a gap between collection periods, state the dates for each sample cohort.*
Data exclusions	*If no data were excluded from the analyses, state so OR if data were excluded, provide the exact number of exclusions and the rationale behind them, indicating whether exclusion criteria were pre-established.*
Non-participation	*State how many participants dropped out/declined participation and the reason(s) given OR provide response rate OR state that no participants dropped out/declined participation.*
Randomization	*If participants were not allocated into experimental groups, state so OR describe how participants were allocated to groups, and if allocation was not random, describe how covariates were controlled.*

Ecological, evolutionary & environmental sciences study design

All studies must disclose on these points even when the disclosure is negative.

Study description	*Briefly describe the study. For quantitative data include treatment factors and interactions, design structure (e.g. factorial, nested, hierarchical), nature and number of experimental units and replicates.*
Research sample	*Describe the research sample (e.g. a group of tagged Passer domesticus, all Stenocereus thurberi within Organ Pipe Cactus National Monument), and provide a rationale for the sample choice. When relevant, describe the organism taxa, source, sex, age range and any manipulations. State what population the sample is meant to represent when applicable. For studies involving existing datasets, describe the data and its source.*
Sampling strategy	*Note the sampling procedure. Describe the statistical methods that were used to predetermine sample size OR if no sample-size calculation was performed, describe how sample sizes were chosen and provide a rationale for why these sample sizes are sufficient.*
Data collection	*Describe the data collection procedure, including who recorded the data and how.*
Timing and spatial scale	*Indicate the start and stop dates of data collection, noting the frequency and periodicity of sampling and providing a rationale for these choices. If there is a gap between collection periods, state the dates for each sample cohort. Specify the spatial scale from which the data are taken*
Data exclusions	*If no data were excluded from the analyses, state so OR if data were excluded, describe the exclusions and the rationale behind them, indicating whether exclusion criteria were pre-established.*
Reproducibility	*Describe the measures taken to verify the reproducibility of experimental findings. For each experiment, note whether any attempts to repeat the experiment failed OR state that all attempts to repeat the experiment were successful.*
Randomization	*Describe how samples/organisms/participants were allocated into groups. If allocation was not random, describe how covariates were controlled. If this is not relevant to your study, explain why.*
Blinding	*Describe the extent of blinding used during data acquisition and analysis. If blinding was not possible, describe why OR explain why blinding was not relevant to your study.*

Did the study involve field work? ☐ Yes ☐ No

Field work, collection and transport

Field conditions	*Describe the study conditions for field work, providing relevant parameters (e.g. temperature, rainfall).*
Location	*State the location of the sampling or experiment, providing relevant parameters (e.g. latitude and longitude, elevation, water depth).*
Access & import/export	*Describe the efforts you have made to access habitats and to collect and import/export your samples in a responsible manner and in compliance with local, national and international laws, noting any permits that were obtained (give the name of the issuing authority, the date of issue, and any identifying information).*
Disturbance	*Describe any disturbance caused by the study and how it was minimized.*

Reporting for specific materials, systems and methods

We require information from authors about some types of materials, experimental systems and methods used in many studies. Here, indicate whether each material, system or method listed is relevant to your study. If you are not sure if a list item applies to your research, read the appropriate section before selecting a response.

Materials & experimental systems

n/a	Involved in the study
☐	☒ Antibodies
☐	☒ Eukaryotic cell lines
☒	☐ Palaeontology and archaeology
☒	☐ Animals and other organisms
☒	☐ Clinical data
☒	☐ Dual use research of concern
☒	☐ Plants

Methods

n/a	Involved in the study
☐	☒ ChIP-seq
☒	☐ Flow cytometry
☒	☐ MRI-based neuroimaging

Antibodies

Antibodies used

In this study, the primary antibody was used at a concentration of 0.5 µg (as mentioned in the Methods section), and the dilution details are provided below.
H3K27me3: Diagenode, Cat. No: C15410195, Lot. No: A0824D Clone name: NA, Dilution: 1:200, host: rabbit.
H3K27ac: abcam, Cat. No: ab4729, Lot. No: GR3442886-1, Clone name: NA, Dilution: 1:200, host: rabbit.
Biotin: Cell Signaling Technology, Cat. No: 5597, Lot. No: 1, Clone name: NA, Dilution: 1:30 (scEpichem), Dilution: 1:250 (IF) , host: rabbit.
Donkey anti-Rabbit-Alexa 488: Life, Cat. No. A32790, Clone name:NA, Dilution: 1:500, host: donkey.
Donkey anti-Rabbit-Alexa 555: Life, Cat. No. A31572, Clone name:NA, Dilution: 1:500, host: donkey.
Goat anti-Rabbit-Alexa 488: Cell Signaling Technology, Cat. No. 4412S, Clone name:NA, Dilution: 1:500, host: goat.

Validation

All primary antibodies are validated by the manufacturer's website as shown below:
H3K27me3: Polyclonal antibody raised in rabbit against the region of histone H3 containing the trimethylated lysine 27 & has been validated in ChIP/ChIP-seq, CUT&TAG, ELISA, IHC-P, Western Blotting, Immunofluorescence: https://www.diagenode.com/en/p/h3k27me3-polyclonal-antibody-premium-sample-size-10-ug
H3K27ac: Rabbit polyclonal to Histone H3 (acetyl K27) & has been validated in ICC/IF, WB, IHC-P, ChIP, PepArr: https://www.abcam.com/products/primary-antibodies/histone-h3-acetyl-k27-antibody-chip-grade-ab4729.html
Biotin: This anti-biotin antibody was raised against Biotin & has been validated in WB, IP, IHC, CUT&TAG, CUT&RUN, DB, eCLIP, IF: https://www.cellsignal.cn/products/secondary-antibodies/anti-biotin-d5a7-rabbit-mab/5597
Secondary antibodies offer increased versatility enabling users to use many detection systems (e.g. HRP, AP, fluorescence). They can also provide greater sensitivity through signal amplification as multiple secondary antibodies can bind to a single primary antibody.
Donkey anti-Rabbit-Alexa 488/555 secondary antibody: Anti-Rabbit secondary antibodies are affinity-purified antibodies with well characterized specificity for rabbit immunoglobulins and are useful in the detection, sorting or purification of its specified target. The antibody has been validated for specificity and application by the manufacturer: https://www.thermofisher.cn/cn/zh/antibody/product/Donkey-anti-Rabbit-IgG-H-L-Highly-Cross-Adsorbed-Secondary-Antibody-Polyclonal/A32790
Donkey anti-Rabbit-Alexa 555 secondary antibody: These donkey anti-rabbit IgG (H+L)whole secondary antibodies have been affinity-purified and show minimum cross-reactivity. Cross-adsorption or pre-adsorption is a purification step to increase specificity of the antibody resulting in higher sensitivity and less background staining. The antibody has been validated for specificity and application by the manufacturer: https://www.thermofisher.cn/cn/zh/antibody/product/Donkey-anti-Rabbit-IgG-H-L-Highly-Cross-Adsorbed-Secondary-Antibody-Polyclonal/A-31572
Goat anti-Rabbit-Alexa 488 secondary antibody: Anti-Rabbit IgG (H+L) F(ab')2 Fragment was conjugated to Alexa Fluor® 488 fluorescent dye under optimal conditions and formulated at 2 mg/ml. This F(ab')2 fragment product results in less non-specific binding, as it lacks the Fc domain that can bind to the cells with Fc receptors. The antibody has been validated for specificity and application by the manufacturer: https://www.cellsignal.cn/products/secondary-antibodies/anti-rabbit-igg-h-l-f-ab-2-fragment-alexa-fluor-488-conjugate/4412

Eukaryotic cell lines

Policy information about cell lines and Sex and Gender in Research

Cell line source(s)	Mouse embryonic stem cells (mESCs) (original from Novus Biologicals, NBP1-41162) provided by H. Deng, Peking University, and maintained in this lab; HGC27 cell line (original from ECACC, CVCL_1279) and SNU16 cell line (original from ATCC, CRL-5974) provided by S. Shu, Peking University Cancer Hospital & Institute, and maintained in this lab; Human K562 erythroleukemia cell line (original from ATCC, CCL-243) maintained in this lab.
Authentication	Cells are evaluated for a typical round shape ESCs morphology with small and tightly packed cells, and a high nucleus/cytoplasm ratio and prominent nucleoli, and stained positively for ESCs marker, OCT4.
Mycoplasma contamination	All the cell lines are negative for Mycoplasma contamination
Commonly misidentified lines (See ICLAC register)	No commonly misidentified cell lines were used.

Palaeontology and Archaeology

Specimen provenance	*Provide provenance information for specimens and describe permits that were obtained for the work (including the name of the issuing authority, the date of issue, and any identifying information). Permits should encompass collection and, where applicable, export.*
Specimen deposition	*Indicate where the specimens have been deposited to permit free access by other researchers.*
Dating methods	*If new dates are provided, describe how they were obtained (e.g. collection, storage, sample pretreatment and measurement), where they were obtained (i.e. lab name), the calibration program and the protocol for quality assurance OR state that no new dates are provided.*

☐ Tick this box to confirm that the raw and calibrated dates are available in the paper or in Supplementary Information.

Ethics oversight	*Identify the organization(s) that approved or provided guidance on the study protocol, OR state that no ethical approval or guidance was required and explain why not.*

Note that full information on the approval of the study protocol must also be provided in the manuscript.

Animals and other research organisms

Policy information about studies involving animals; ARRIVE guidelines recommended for reporting animal research, and Sex and Gender in Research

Laboratory animals	The study did not involve laboratory animals.
Wild animals	The study did not involve wild animals.
Reporting on sex	The study did not involve wild animals.
Field-collected samples	We analysed specimens derived from two colorectal cancer patients.
Ethics oversight	This study was approved by the Research Ethics Committee of both Peking University First Hospital and Peking University People's Hospital

Note that full information on the approval of the study protocol must also be provided in the manuscript.

Clinical data

Policy information about clinical studies

All manuscripts should comply with the ICMJE guidelines for publication of clinical research and a completed CONSORT checklist must be included with all submissions.

Clinical trial registration	*Provide the trial registration number from ClinicalTrials.gov or an equivalent agency.*
Study protocol	*Note where the full trial protocol can be accessed OR if not available, explain why.*
Data collection	*Describe the settings and locales of data collection, noting the time periods of recruitment and data collection.*
Outcomes	*Describe how you pre-defined primary and secondary outcome measures and how you assessed these measures.*

Dual use research of concern

Policy information about dual use research of concern

Hazards

Could the accidental, deliberate or reckless misuse of agents or technologies generated in the work, or the application of information presented in the manuscript, pose a threat to:

No	Yes	
☐	☐	Public health
☐	☐	National security
☐	☐	Crops and/or livestock
☐	☐	Ecosystems
☐	☐	Any other significant area

Experiments of concern

Does the work involve any of these experiments of concern:

No	Yes	
☐	☐	Demonstrate how to render a vaccine ineffective
☐	☐	Confer resistance to therapeutically useful antibiotics or antiviral agents
☐	☐	Enhance the virulence of a pathogen or render a nonpathogen virulent
☐	☐	Increase transmissibility of a pathogen
☐	☐	Alter the host range of a pathogen
☐	☐	Enable evasion of diagnostic/detection modalities
☐	☐	Enable the weaponization of a biological agent or toxin
☐	☐	Any other potentially harmful combination of experiments and agents

Plants

Seed stocks	*Report on the source of all seed stocks or other plant material used. If applicable, state the seed stock centre and catalogue number. If plant specimens were collected from the field, describe the collection location, date and sampling procedures.*
Novel plant genotypes	*Describe the methods by which all novel plant genotypes were produced. This includes those generated by transgenic approaches, gene editing, chemical/radiation-based mutagenesis and hybridization. For transgenic lines, describe the transformation method, the number of independent lines analyzed and the generation upon which experiments were performed. For gene-edited lines, describe the editor used, the endogenous sequence targeted for editing, the targeting guide RNA sequence (if applicable) and how the editor was applied.*
Authentication	*Describe any authentication procedures for each seed stock used or novel genotype generated. Describe any experiments used to assess the effect of a mutation and, where applicable, how potential secondary effects (e.g. second site T-DNA insertions, mosiacism, off-target gene editing) were examined.*

ChIP-seq

Data deposition

☒ Confirm that both raw and final processed data have been deposited in a public database such as GEO.

☒ Confirm that you have deposited or provided access to graph files (e.g. BED files) for the called peaks.

Data access links<br>*May remain private before publication.*	The raw sequence data reported in this paper have been deposited in the Genome Sequence Archive in National Genomics Data Center, China National Center for Bioinformation/Beijing Institute of Genomics, Chinese Academy of Sciences (accession number: GSA-Human:HRA005220, HRA005219 https://ngdc.cncb.ac.cn/gsa-human). The raw sequence data reported in this paper have been deposited in the Genome Sequence Archive (Genomics, Proteomics & Bioinformatics 2021) in National Genomics Data Center (Nucleic Acids Res 2022), China National Center for Bioinformation / Beijing Institute of Genomics, Chinese Academy of Sciences (GSA: CRA012132) that are publicly accessible at https://ngdc.cncb.ac.cn/gsa.
Files in database submission	Bulk_BRD4_K562.bam<br>Bulk_JQ1_btn_K562_rep1.bam<br>Bulk_JQ1_btn_K562_rep2.bam<br>Bulk_BRD4_HGC27.bam

Bulk_JQ1_btn_HGC27_rep1.bam
Bulk_JQ1_btn_HGC27_rep2.bam
sc_Single_omic_JQ1_K562.merge.bam
sc_Double_omic_JQ1_K562.merge.bam
sc_Double_omic_JQ1_HGC27.merge.bam
sc_Double_omic_JQ1_v6.5.merge.bam
sc_Double_omic_H3K27me3_K562.merge.bam
sc_Double_omic_H3K27me3_HGC27.merge.bam
sc_Double_omic_H3K27me3_v6.5.merge.bam
sc_Tri_omic_ATAC_K562_agg.bam
sc_Tri_omic_JQ1_K562_agg.bam
sc_Tri_omic_BRD4_K562_agg.bam
P20_bulk_THZ1-btn_rep1.bam
P20_bulk_THZ1-btn_rep2.bam
P20_bulk_DOX-btn_rep1.bam
P20_bulk_DOX-btn_rep2.bam
P1201_P20_Tri_omic_BRD4_merge.bam
P1201_P20_Tri_omic_JQ1-btn_merge.bam
P1201_P20_Tri_omic_ATAC_merge.bam
P1201_P20_Double_omic_H3K27ac_merge.bam
P1201_P20_Double_omic_DOX-btn_merge.bam
P1201_P20_Double_omic_JQ1-btn_merge.bam
P1201_P20_Double_omic_THZ1-btn_merge.bam
P1201_Day0_Double_omic_THZ1-btn_merge.bam
P1201_Day0_Double_omic_H3K27ac_THZ1-btn_merge.bam
P1201_Day0_Double_omic_JQ1-btn_merge.bam
P1201_Day0_Double_omic_H3K27ac_JQ1-btn_merge.bam
P1201_Day0_Double_omic_DOX-btn_merge.bam
P1201_Day0_Double_omic_H3K27ac_DOX-btn_merge.bam
P20_Day0_Double_omic_THZ1-btn_merge.bam
P20_Day0_Double_omic_H3K27ac_THZ1-btn_merge.bam
P20_Day0_Double_omic_JQ1-btn_merge.bam
P20_Day0_Double_omic_H3K27ac_JQ1-btn_merge.bam
P20_Day0_Double_omic_DOX-btn_merge.bam
P20_Day0_Double_omic_H3K27ac_DOX-btn_merge.bam
P1201_Day3_Double_omic_THZ1-btn_merge.bam
P1201_Day3_Double_omic_H3K27ac_THZ1-btn_merge.bam
P1201_Day3_Double_omic_JQ1-btn_merge.bam
P1201_Day3_Double_omic_H3K27ac_JQ1-btn_merge.bam
P1201_Day3_Double_omic_DOX-btn_merge.bam
P1201_Day3_Double_omic_H3K27ac_DOX-btn_merge.bam
P20_Day3_Double_omic_THZ1-btn_merge.bam
P20_Day3_Double_omic_H3K27ac_THZ1-btn_merge.bam
P20_Day3_Double_omic_JQ1-btn_merge.bam
P20_Day3_Double_omic_H3K27ac_JQ1-btn_merge.bam
P20_Day3_Double_omic_DOX-btn_merge.bam
P20_Day3_Double_omic_H3K27ac_DOX-btn_merge.bam
P1201_Day5_Double_omic_THZ1-btn_merge.bam
P1201_Day5_Double_omic_H3K27ac_THZ1-btn_merge.bam
P1201_Day5_Double_omic_JQ1-btn_merge.bam
P1201_Day5_Double_omic_H3K27ac_JQ1-btn_merge.bam
P1201_Day5_Double_omic_DOX-btn_merge.bam
P1201_Day5_Double_omic_H3K27ac_DOX-btn_merge.bam
P20_Day5_Double_omic_THZ1-btn_merge.bam
P20_Day5_Double_omic_H3K27ac_THZ1-btn_merge.bam
P20_Day5_Double_omic_JQ1-btn_merge.bam
P20_Day5_Double_omic_H3K27ac_JQ1-btn_merge.bam
P20_Day5_Double_omic_DOX-btn_merge.bam
P20_Day5_Double_omic_H3K27ac_DOX-btn_merge.bam

Genome browser session (e.g. UCSC)

no longer applicable

Methodology

Replicates

Two biological replicates for scEpi-Chem libraries.

Sequencing depth

All the libraries were sequenced by PE150. Supplementary Table 3 provides the details.

Antibodies

H3K27me3: Diagenode, Cat. No: C15410195, Lot. No: A0824D Clone name: NA, Dilution: 1:200.
H3K27ac: abcam, Cat. No: ab4729, Lot. No: GR3442886-1, Clone name: NA, Dilution: 1:200.
Biotin: Cell Signaling Technology, Cat. No: 5597, Lot. No: 1, Clone name: NA, Dilution: 1:30 (scEpichem), Dilution: 1:250 (IF).
Donkey anti-Rabbit-Alexa 488: Life, Cat. No. A32790, Clone name:NA, Dilution: 1:500.
Donkey anti-Rabbit-Alexa 555: Life, Cat. No. A31572, Clone name:NA, Dilution: 1:500.
Goat anti-Rabbit-Alexa 488: Cell Signaling Technology, Cat. No. 4412S, Clone name:NA, Dilution: 1:500.

Peak calling parameters	Peaks were identified using MACS2 with the parameter setting (--broad).
Data quality	We evaluated the data quality by track view, calculating the correlation of different groups and etc.
Software	FastQC (v 0.11.5), Bowtie2 (v 2.2.9), Samtools (v 1.9), Picard (v 2.2.4), MACS2 (v 2.1.1), Deeptools (v 3.5.1), Seurat (v 4.3.0) , cisTopic (v 0.3.0).

Flow Cytometry

Plots

Confirm that:

- [ ] The axis labels state the marker and fluorochrome used (e.g. CD4-FITC).
- [ ] The axis scales are clearly visible. Include numbers along axes only for bottom left plot of group (a 'group' is an analysis of identical markers).
- [ ] All plots are contour plots with outliers or pseudocolor plots.
- [ ] A numerical value for number of cells or percentage (with statistics) is provided.

Methodology

Sample preparation	*Describe the sample preparation, detailing the biological source of the cells and any tissue processing steps used.*
Instrument	*Identify the instrument used for data collection, specifying make and model number.*
Software	*Describe the software used to collect and analyze the flow cytometry data. For custom code that has been deposited into a community repository, provide accession details.*
Cell population abundance	*Describe the abundance of the relevant cell populations within post-sort fractions, providing details on the purity of the samples and how it was determined.*
Gating strategy	*Describe the gating strategy used for all relevant experiments, specifying the preliminary FSC/SSC gates of the starting cell population, indicating where boundaries between "positive" and "negative" staining cell populations are defined.*

- [ ] Tick this box to confirm that a figure exemplifying the gating strategy is provided in the Supplementary Information.

Magnetic resonance imaging

Experimental design

Design type	*Indicate task or resting state; event-related or block design.*
Design specifications	*Specify the number of blocks, trials or experimental units per session and/or subject, and specify the length of each trial or block (if trials are blocked) and interval between trials.*
Behavioral performance measures	*State number and/or type of variables recorded (e.g. correct button press, response time) and what statistics were used to establish that the subjects were performing the task as expected (e.g. mean, range, and/or standard deviation across subjects).*

Acquisition

Imaging type(s)	*Specify: functional, structural, diffusion, perfusion.*
Field strength	*Specify in Tesla*
Sequence & imaging parameters	*Specify the pulse sequence type (gradient echo, spin echo, etc.), imaging type (EPI, spiral, etc.), field of view, matrix size, slice thickness, orientation and TE/TR/flip angle.*
Area of acquisition	*State whether a whole brain scan was used OR define the area of acquisition, describing how the region was determined.*

Diffusion MRI ☐ Used ☐ Not used

Preprocessing

Preprocessing software	*Provide detail on software version and revision number and on specific parameters (model/functions, brain extraction, segmentation, smoothing kernel size, etc.).*
Normalization	*If data were normalized/standardized, describe the approach(es): specify linear or non-linear and define image types used for*

Normalization

transformation OR indicate that data were not normalized and explain rationale for lack of normalization.

Normalization template

Describe the template used for normalization/transformation, specifying subject space or group standardized space (e.g. original Talairach, MNI305, ICBM152) OR indicate that the data were not normalized.

Noise and artifact removal

Describe your procedure(s) for artifact and structured noise removal, specifying motion parameters, tissue signals and physiological signals (heart rate, respiration).

Volume censoring

Define your software and/or method and criteria for volume censoring, and state the extent of such censoring.

Statistical modeling & inference

Model type and settings

Specify type (mass univariate, multivariate, RSA, predictive, etc.) and describe essential details of the model at the first and second levels (e.g. fixed, random or mixed effects; drift or auto-correlation).

Effect(s) tested

Define precise effect in terms of the task or stimulus conditions instead of psychological concepts and indicate whether ANOVA or factorial designs were used.

Specify type of analysis: ☐ Whole brain ☐ ROI-based ☐ Both

Statistic type for inference

(See Eklund et al. 2016)

Specify voxel-wise or cluster-wise and report all relevant parameters for cluster-wise methods.

Correction

Describe the type of correction and how it is obtained for multiple comparisons (e.g. FWE, FDR, permutation or Monte Carlo).

Models & analysis

n/a	Involved in the study
☐	☐ Functional and/or effective connectivity
☐	☐ Graph analysis
☐	☐ Multivariate modeling or predictive analysis

Functional and/or effective connectivity

Report the measures of dependence used and the model details (e.g. Pearson correlation, partial correlation, mutual information).

Graph analysis

Report the dependent variable and connectivity measure, specifying weighted graph or binarized graph, subject- or group-level, and the global and/or node summaries used (e.g. clustering coefficient, efficiency, etc.).

Multivariate modeling and predictive analysis

Specify independent variables, features extraction and dimension reduction, model, training and evaluation metrics.
